# Landscape of mSWI/SNF chromatin remodeling complex perturbations in neurodevelopmental disorders

**DOI:** 10.1038/s41588-023-01451-6

**Published:** 2023-07-27

**Authors:** Alfredo M. Valencia, Akshay Sankar, Pleuntje J. van der Sluijs, F. Kyle Satterstrom, Jack Fu, Michael E. Talkowski, Samantha A. Schrier Vergano, Gijs W. E. Santen, Cigall Kadoch

**Affiliations:** 1 Dana-Farber Cancer Institute and Harvard Medical School, Boston, MA USA; 2 Chemical Biology Program, Harvard University, Cambridge, MA USA; 3 Broad Institute of MIT and Harvard, Cambridge, MA USA; 4 Department of Clinical Genetics, Leiden University Medical Center, Leiden, the Netherlands; 5 Stanley Center for Psychiatric Research, Broad Institute of MIT and Harvard, Cambridge, MA USA; 6 Massachusetts General Hospital, Boston, MA USA; 7 Children’s Hospital of the King’s Daughters, Norfolk, Virginia USA; 8 Department of Pediatrics, Eastern Virginia Medical School, Norfolk, Virginia USA; 9 Howard Hughes Medical Institute, Chevy Chase, MD USA; 10 Present Address: Department of Psychiatry and Behavioral Sciences, Stanford University, Stanford, CA USA; 11 Present Address: Stanford Brain Organogenesis, Wu Tsai Neurosciences Institute, Stanford University, Stanford, CA USA

**Keywords:** Epigenetics, Neuroscience, Epigenetics, Neurological disorders

## Abstract

DNA sequencing-based studies of neurodevelopmental disorders (NDDs) have identified a wide range of genetic determinants. However, a comprehensive analysis of these data, in aggregate, has not to date been performed. Here, we find that genes encoding the mammalian SWI/SNF (mSWI/SNF or BAF) family of ATP-dependent chromatin remodeling protein complexes harbor the greatest number of de novo missense and protein-truncating variants among nuclear protein complexes. Non-truncating NDD-associated protein variants predominantly disrupt the cBAF subcomplex and cluster in four key structural regions associated with high disease severity, including mSWI/SNF-nucleosome interfaces, the ATPase-core ARID-armadillo repeat (ARM) module insertion site, the Arp module and DNA-binding domains. Although over 70% of the residues perturbed in NDDs overlap with those mutated in cancer, ~60% of amino acid changes are NDD-specific. These findings provide a foundation to functionally group variants and link complex aberrancies to phenotypic severity, serving as a resource for the chromatin, clinical genetics and neurodevelopment communities.

## Abstract (web summary)

Genes encoding members of mammalian SWI/SNF (BAF) complexes are frequently mutated in individuals with neurodevelopmental disorders (NDDs). Mutant NDD residues include some unique to NDD and those shared with human cancers, impacting key structural hubs.

## Main

Sequencing studies have revealed extensive involvement of chromatin regulatory processes in a range of human diseases, with frequent mutations in the genes encoding proteins that govern chromatin architecture^1–4^. Four families of multi-subunit ATP-dependent chromatin remodeling complexes (SWI/SNF, ISWI, CHD and INO80) modulate chromatin topology and gene expression by mobilizing their nucleosome substrates^5^. Recent advances in cryo-electron microscopy (cryo-EM), cross-linking mass spectrometry and homology modeling have begun to uncover the three-dimensional (3D) structure and modes of nucleosome substrate engagement of these large heterogeneous entities, informing mechanistic studies^6^.

Mutations in the genes encoding mammalian SWI/SNF (mSWI/SNF) chromatin remodeling complex are found in over 20% of cases in cancer, which has stimulated a range of basic and translational efforts over the past several years^7–9^. A wealth of mutational data of neurodevelopmental disorders (NDDs), such as intellectual disability and autism spectrum disorders, has also recently emphasized a high mutational burden of chromatin regulatory genes in NDD, presenting an opportunity to dissect the molecular underpinnings and to inform potential strategies to remedy the comorbid issues associated with these disorders^2,10–14^.

Most cancer-associated mSWI/SNF mutations result in subunit deletions or gene silencing, which has presented the field with opportunities to understand the impact of full subunit losses and the impact on complex disassembly^15–18^. NDD-associated mSWI/SNF genetic variants present particularly unique opportunities for functional dissection, in that 1) mutations are often missense, affecting single amino acids and clustering in defined domains within subunits; 2) mutations are predominantly heterozygous, underscoring the high degree of dosage sensitivity; and 3) mutations are often found as the sole genetic cause of these disorders. Furthermore, for trios in which parents’ genetic information is available, mSWI/SNF gene variants are predominantly de novo (absent in parents), indicating their causative role^19–21^. Together, these features enable functional assignment and prioritization for specific subunit domains and even individual protein residues. Identifying and mechanistically defining these variants will be critical for the assignment of specific chromatin remodeling complex functions and, ultimately, informing therapeutic approaches for a range of human diseases driven by mSWI/SNF complex disruption.

Here, we sought to comprehensively catalog and integrate mSWI/SNF complex sequence variants across a diverse collection of datasets, including the Simon’s Foundation Research Initiative (SFARI) (Simons Foundation Powering Autism Research for Knowledge (SPARK), Simons Searchlight Collection–Autism Sequencing Consortium (SSC-ASC)), the Deciphering Developmental Disorders project (DDD), the DECIPHER database^22^, ClinVar^23^, the Leiden Open Variation Database (LOVD)^24^, de novo sequence variants from the literature (as performed in McRae et al. (https://github.com/jeremymcrae/dnm_cohorts)^3,25–39^), NDD-associated mSWI/SNF sequence variants from the literature^3,19–21,35,40–81^ and 85 previously unreported NDD-associated mSWI/SNF cases, including 72 novel variants, focused on protein coding mutations stemming from single-nucleotide variants (SNVs) and small insertions/deletions (indels) (Supplementary Table 1). These analyses encompass 2,539 total cases of which the majority (67.1%, *n* = 1,703) result in missense and in-frame indels that collectively reveal 1,204 unique variants.

## Results

### Chromatin remodelers carry a high mutational burden in NDDs

**Fig. 1:**
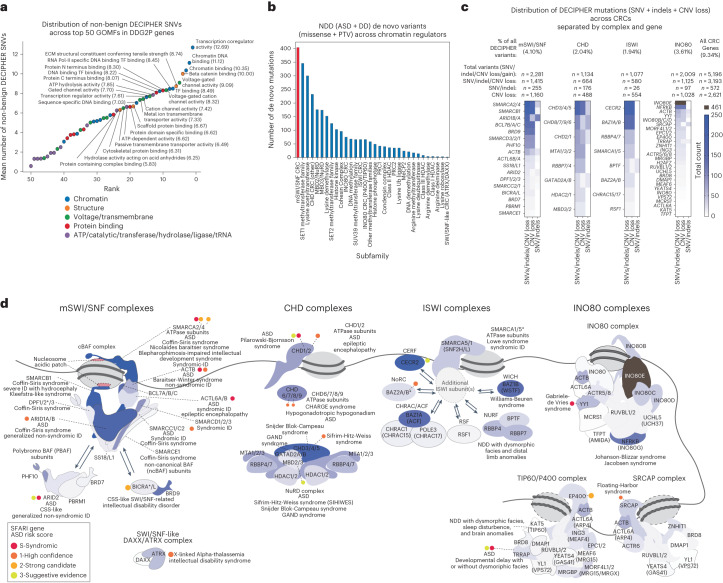
Genes encoding chromatin regulatory complexes represent the most frequently mutated gene classes in human NDDs. **a**, Scatterplot of the average numbers of non-benign SNVs in DECIPHER corresponding to the top 50 GOMF gene sets enriched in DDG2P developmental disorder-associated genes, ranked by the mutational burden of each gene set. **b**, Bar graph depicting the total number of NDD-associated missense and protein truncating variants (PTVs) for a curated list of chromatin regulatory and epigenetic gene sets, ranked by mutational burden of each gene set in autism spectrum disorders and developmental disorders (ASD + DD) from the Simons Foundation Research Initiative (SFARI) datasets (SPARK: Simons Foundation Powering Autism Research + SSC-ASC: Simons Searchlight Collection–Autism Sequencing Consortium, and DDD: Deciphering developmental disorders studies). The mSWI/SNF chromatin remodeling complex gene set is emphasized in red. **c**, Heatmaps depicting the mutational frequency for genes encoding members of the SWI/SNF, CHD, ISWI, and INO80 complex families in DECIPHER. Total number of variants (including copy-number variant (CNV) gain, copy number variant loss (CNV loss), single nucleotide variant (SNV) and indel mutational frequencies are indicated. Percentage of total DECIPHER sequence variants are indicated for each chromatin remodeling complex family (top). **d**, Cartoon representations of the four classes of chromatin remodelers (SWI/SNF, CHD, ISWI and INO80) and respective subcomplex or related complex associations, colored by CNV loss/SNV/indel variation frequency from panel **c**. Interchangeable subunit paralogs are colored by their combined mutational frequency. Autism spectrum disorder (ASD) risk score (SFARI) and developmental disorder associations curated from literature and OMIM (Online Mendelian Inheritance in Man, a catalog of human genes and genetic disorders; https://www.omim.org/) are indicated. Asterisk (*) indicates paralog implicated in NDD. Where possible, cartoons were based on 3D structural data available from human and yeast structures; ovals are used in in lieu of structural cartoons for components lacking structural data.

**Extended Data Fig. 1:**
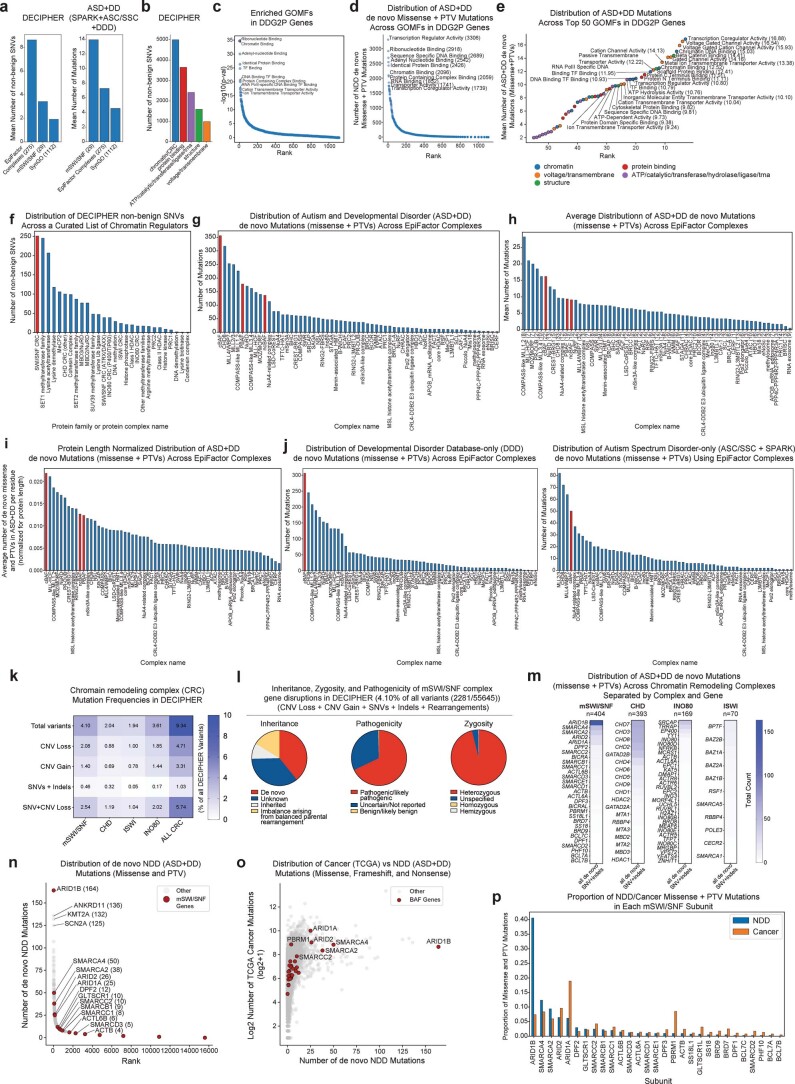
SWI/SNF complex genes are among the most frequently mutated genes in human NDD. **a**, Bar charts depicting mean number of non-benign SNVs in DECIPHER and ASD+DD across gene sets indicated. **b**, Bar graph summarizing the number of non-benign DECIPHER SNVs across top 5 categories from Fig. 1a. **c–e**, Rank plots depicting GOMF gene sets in (c) DDG2P, (d) ranked by total number of ASD+DD de novo missense variants, (e) ranked by mutation frequency, top 50 GOMFs. **f–j**, Bar charts showing distribution of variants across sets indicated in each title. mSWI/SNF or cBAF, PBAF, and ncBAF are highlighted in red. **k**, Heatmap summarizing DECIPHER database mutational frequency for each chromatin remodeling complex separated by variant type (all variants, copy number variants (CNV), and SNVs/indels). **l**, Pie charts showing inheritance, pathogenicity, and zygosity breakdown of all mSWI/SNF complex variants from DECIPHER. **m**, Heatmaps depicting the mutational frequency of chromatin remodeling genes in SWI/SNF, CHD, ISWI, and INO80 complex family classes in the ASD+DD dataset. Total number of SNV and indel variants per protein complex family indicated. **n**, Scatterplot of the total number of de novo missense and PTVs in ASD+DD for all genes ranked by the mutational burden of each gene. mSWI/SNF genes are shown in red. **o**, Scatterplot of the log normalized total number of cancer missense, frameshift, and nonsense mutations in the TCGA MC3 PanCancer dataset versus the total number of NDD de novo missense and PTVs in ASD+DD datasets. mSWI/SNF genes shown in red. **p**, Grouped bar graph of the proportion of NDD (blue) and cancer (orange) missense and PTV mutations across all mSWI/SNF genes sorted by decreasing NDD mutational proportion.

Single amino acid mutations and protein-truncating variants (PTVs) in chromatin regulatory genes are pathogenic for a variety of NDDs, including syndromic and non-syndromic intellectual disabilities and autism spectrum disorders^3^, but their relative prevalence remains undefined. We collated and analyzed all SNVs and small indels reported in DECIPHER (DatabasE of genomiC varIation and Phenotype in Humans using Ensembl Resources)^22^ (https://www.deciphergenomics.org/), a repository of clinical and genetic information on individuals with developmental disorders. Remarkably, we found that epigenetic and chromatin-related genes (EpiFactor gene list, Supplementary Table 2)^82^ were more frequently mutated than synapse-related genes (SynGO gene list, Supplementary Table 2)^83^, which are known to be highly implicated in NDDs (Extended Data Fig. 1a). By examining the top 50 Gene Ontology molecular functions (GOMFs) of genes in the Development Disorder Genotype–Phenotype Database (DDG2P), we found that top-ranked disrupted processes were enriched for transcription- and chromatin-related processes, with transcription and chromatin binding terms ranking highest among them (Fig. 1a and Extended Data Fig. 1b,c). Performing this analysis with variants identified from the SFARI Autism Spectrum Disorder (ASD) SPARK, SSC-ASC and developmental disorder (DD) DDD study datasets (ASD + DD) revealed similar results, including transcription-, synapse- and chromatin-related GOMFs (that is, 1: transcriptional coregulator activity, 2: voltage-gated channel activity, 3: voltage-gated cation channel activity and 4: chromatin DNA binding) (Extended Data Fig. 1a,c–e). We then analyzed de novo missense and PTV frequencies from ASD + DD datasets by protein complex associations and by chromatin regulatory activity, which revealed the greatest number of variants occurred in SWI/SNF chromatin remodeling complex genes (protein complex, *n* = 404 sequence variants, rank 1), followed by SET1 methyltransferase family (protein family, *n* = 346, rank 2), lysine acetyltransferases (protein family, *n* = 300, rank 3) and CHD chromatin remodeling complex genes (protein complex, *n* = 232, rank 4) (Fig. 1b and Supplementary Table 2). This result was consistent using DECIPHER data (Extended Data Fig. 1f) and chromatin-related protein complexes from EpiFactor using ASD + DD data (Extended Data Fig. 1g). Of note, several histone modifying complexes, including the histone–lysine *N*-methyltransferase (KMT2 or MLL) family of complexes, the histone acetyltransferase MOZ/MORF complexes and Polycomb repressive deubiquitinase (PR-DUB) complexes had a greater average of mutations when normalized by gene set size, owing to lower numbers of defined components relative to mSWI/SNF complexes (average ~6 components versus ~19 components for mSWI/SNF) (Extended Data Fig. 1h and Supplementary Table 2). Nevertheless, when normalized by protein length (or gene exon length), cBAF complexes maintained the highest average number of de novo mutations and PTVs compared to all EpiFactor complexes (Extended Data Fig. 1i). Interestingly, separating ASD and DD datasets revealed cBAF was the most frequently mutated gene set in DD but ranked fourth in ASD, potentially suggesting a subtle distinction between ASD-associated variants from SFARI compared to a mixture of ASD and other NDDs reported in the DDD database (Extended Data Fig. 1j).

Expanding our analysis to include copy-number variants in addition to SNVs/indels using DECIPHER, we found that genes encoding all members of mammalian chromatin remodeling complexes (across all families) are implicated in approximately one in ten of all DECIPHER cases (9.34%, 5,196/55,645) (Fig. 1c,d and Extended Data Fig. 1k). The 29 genes encoding the mSWI/SNF complex are affected in the greatest percentage (4.10%, 2,281/55,645), the majority of which are classified as ‘pathogenic’ or ‘likely pathogenic’ (67.9%, 1,548/2,281), 39.2% of which were confirmed de novo and 34.4 % of unknown inheritance (Extended Data Fig. 1l). Many mSWI/SNF genes are also implicated in ASD, as characterized by SFARI database (Fig. 1d)^84^. Notably, genes such as *ARID1B*, *SMARCA4* and *SMARCA2* were among the top mSWI/SNF genes with most de novo missense and PTVs across all ASD + DD cases, with *ARID1B* having the most variants, followed by *ANKRD11, KMT2A*, and *SCN2A* (Extended Data Fig. 1m–n). When including CNV losses and sequence variants from DECIPHER, the top mSWI/SNF genes implicated were *SMARCB1* and *SMARCA2*, mutations in which cause the most severe phenotypes of mSWI/SNF-related NDDs, CSS and Nicolaides-Baraitser syndrome (NCBRS), respectively^85^ (Fig. 1c). Nevertheless, multiple genes may be disrupted in a given CNV, making genotype-phenotype correlations more challenging to directly assess. As compared to cancer, wherein mutations in mSWI/SNF genes are present in 20.3% of all cases sequenced^86^ (COSMIC: the Catalog of Somatic Mutations in Cancer), specific mSWI/SNF subunits were more frequently mutated in NDD relative to other mSWI/SNF genes. These included *ARID1B*, the paralog of which, *ARID1A*, is among one of the most frequently mutated genes in cancer, *SMARCA4*, and *SMARCA2* (Extended Data Fig. 1o). Notably, genes encoding PBAF and ncBAF components such as *PBRM1, ARID2, BICRAL* (*GLTSCR1L*) and others were found to be more frequently mutated in cancer than in NDD (Extended Data Fig. 1p). As the most frequently mutated chromatin remodeler in NDDs and cancer, the remainder of this Analysis is centered on the mSWI/SNF family of chromatin remodeling complexes.

### mSWI/SNF NDD variants accumulate in functional domains

**Fig. 2:**
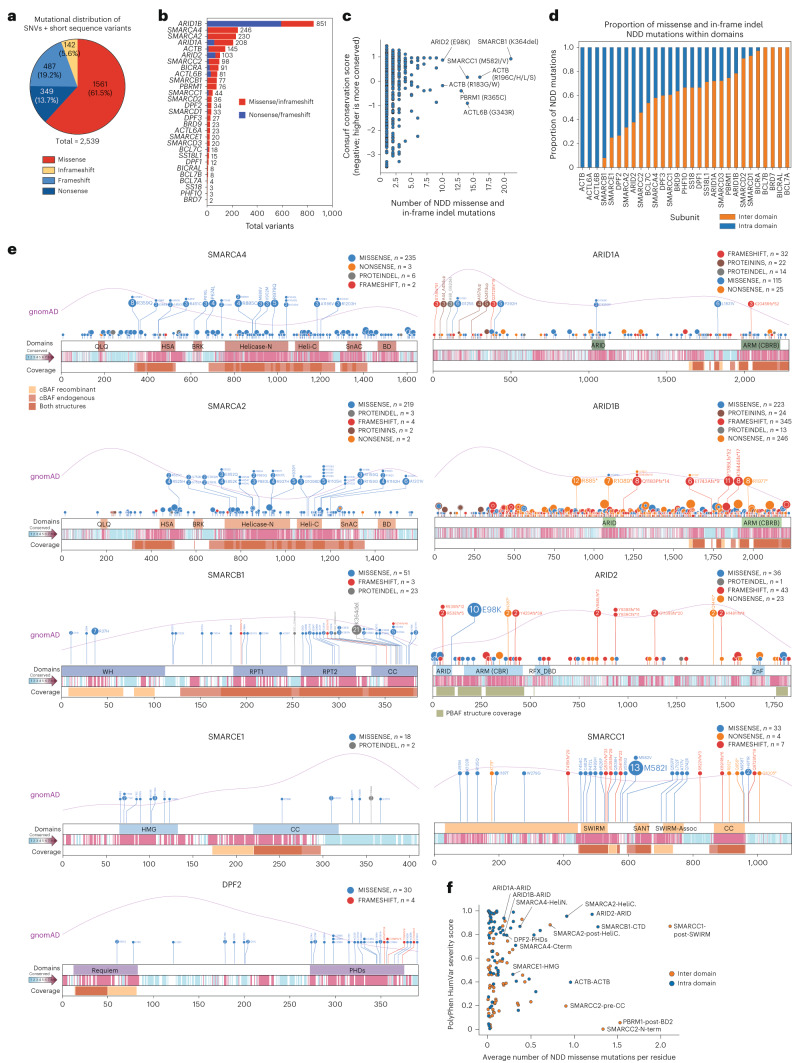
Analysis of NDD-associated SNV and indel mutations in mSWI/SNF complex components. **a**, Pie chart reflecting the distribution of *n* = 2,539 mSWI/SNF NDD-associated SNV and in-frame indel mutations from an integrated dataset containing data from SPARK, SSC-ASC, DDD, DECIPHER, ClinVar, LOVD, literature curation and novel variants reported in this study. **b**, Bar chart summarizing total NDD-associated missense/in-frame deletions and insertions (red) and nonsense/frameshift-inducing mutations (blue) across all mSWI/SNF genes. **c**, Scatterplot of the negative-normalized ConSurf conservation score versus the mutational recurrence at each mSWI/SNF complex subunit residue for NDD missense and in-frame variants in the integrated dataset. Highly conserved and highly mutated positions are labeled. **d**, Stacked bar chart summarizing proportion of NDD-associated missense and in-frame insertion/deletion variants falling within (intra, blue) or outside (inter, orange) of mSWI/SNF subunit domains in the integrated dataset. Domains annotated by PFAM, UniProtKB, manual curation, and structurally resolved domains (see also Supplementary Table 3). **e**, Lollipop plots of NDD mutations in the integrated dataset across protein domain schematics of ARID1A/B, ARID2, SMARCA2/4, SMARCB1, SMARCC1, SMARCE1, and DPF2 subunits generated with Protein Paint. Missense (blue), nonsense (orange), frameshift (red), in-frame deletions (gray) and insertions (brown) are shown. Kernel density estimates (relative frequency distribution) of gnomAD missense mutations (purple line) are overlaid. Domain annotations informed by PFAM, UniProtKB, manual curation, or by structurally resolved domains are indicated. ConSurf conservation scores are shown in a cyan-white-magenta heatmap in increasing conservation order, and structural coverages of the nucleosome core particle (NCP)-bound human cBAF (light orange, PDB: 6LTJ), endogenous human cBAF-NCP bound (red, PDBDEV00000056), and by both structures (brown). Structural coverage for the NCP-bound PBAF complex is also shown for ARID2 (light green, PDB:7VDV). **f**, PolyPhen HumVar predicted phenotypic severity score and missense mutational recurrence of mSWI/SNF gene mutations from the integrated dataset in intra (blue) and inter (orange) domains.

**Extended Data Fig. 2:**
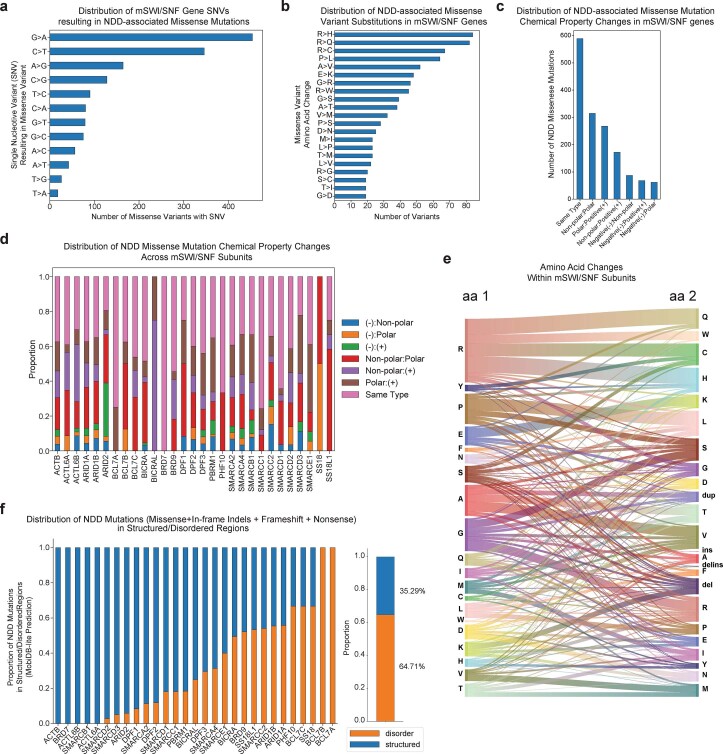
Characteristics of NDD-associated single-residue amino acid perturbations in mSWI/SNF components. **a**, Distribution of single-nucleotide variants (SNVs) found in NDD-associated missense mutations of mSWI/SNF family genes (Supplementary Table 1) in the integrated dataset (n=2539). **b**, Horizontal bar graphs of the top 20 amino acid missense substitutions in the integrated dataset (Supplementary Table 1). **c**, Bar chart characterizing amino acid chemical property changes upon missense mutation for NDD-associated variants in the integrated dataset. **d**, Stacked bar graphs of the distribution of amino acid substitution chemical property changes in NDD-associated missense mutations in the integrated dataset. **e**, Sankey diagram of the distribution of NDD-associated missense substitutions in the integrated dataset. Ribbon thickness represents frequency of substitutions in the integrated dataset. **f**, Stacked bar chart summarizing percentage of NDD-associated missense and in-frame indel mutations in the integrated dataset falling within intrinsically disordered (defined by MobiDB-lite) or structured regions for (left) each mSWI/SNF subunit and (right) all mSWI/SNF subunits combined.

To comprehensively examine the full constellation of mSWI/SNF sequence variants in NDD, we combined mSWI/SNF gene mutations from the DECIPHER, ClinVar, LOVD, SFARI SPARK and SSC–ASC datasets and merged these with mutations reported in published literature as well as n = 85 novel, previously unreported cases (Supplementary Table 1). After removing duplicates, variants with a mutant allele frequency of >0.5% in the general population as assessed by gnomAD^87^, and filtering for missense, inframeshift (herein defined as non-frameshift inducing insertions/deletions), frameshift and nonsense variants, we identified 2539 variants in mSWI/SNF genes, 61.5% of which were missense (Fig. 2a). Variants resulted predominantly in missense or inframeshift (67.1%) (Fig. 2a,b), with the exception of *ARID1B* and *ARID2*, for which the majority of variants were nonsense or frameshift (Fig. 2b). The greatest number of missense variants stemmed from G > A and C > T base pair conversions, resulting in a variety of amino acid changes (Extended Data Fig. 2a–e). The most frequently altered residues were Arginine (R), Proline (P), Alanine (A), and Glycine (G), together making up 47% (815/1703) of all missense and inframeshift affected residues in the dataset (Extended Data Fig. 2b–e, Supplementary Table 1). Furthermore, the most common missense amino acid substitution was Arginine to Histidine (Arg>His; R > H), indicating reductions in both the relative size and pKa of the amino acid side chain (Arg pKa 12.48 – His pKa 6.0) (Extended Data Fig. 2e).

A high percentage of missense and indel mSWI/SNF mutations localized to highly conserved regions (53.1% high, 24.7% moderate conservation) (Fig. 2c). Mutations in subunits such as ACTB, ACTL6A/B, DPF2, and SMARCB1 entirely or nearly entirely occurred in intra-domain structured regions, whereas variants in BCL7A/B, PHF10, and ARID1A/B subunits were skewed toward interdomain disordered regions (Fig. 2d, Extended Data Fig. 2f and Supplementary Table 3). Intriguingly, mutations in SMARCA2 clustered in the ATPase/helicase domain, whereas mutations in SMARCA4 were more dispersed throughout the protein, including the structurally unresolved N terminus (Fig. 2e). Interestingly, whereas mutations within the SMARCA2 helicase cause NCBRS, SMARCA2 mutations outside of this domain are implicated in a distinct disorder, blepharophimosis-impaired intellectual disability syndrome^88^. Among mSWI/SNF paralogs, frameshift mutations were more enriched in ARID1B, whereas missense mutations in specific regions were enriched in ARID1A, clustering namely in the ARID DNA-binding domain, the structurally unresolved N terminus and the C-terminal armadillo repeat domain (ARM or core binding region) (Fig. 2e). A possibility underlying this difference is that ARID1A haploinsufficient mutations lead to a more severe phenotype, as suggested by the frequent occurrence of mosaic variants^69^ and further substantiated during the review process by an analysis of fetal cases^89^.

Genotype-phenotype clinical studies have suggested that *ARID1B* truncating mutations are generally linked to the mildest cases of CSS-related intellectual disability, including some individuals without intellectual disability^90^, whereas single amino acid mutations of the SMARCB1 protein are correlated with the most severe cognitive impairment and growth delay in CSS^21,69,85^. SMARCA2-ATPase mutations result in severe intellectual disability cases of NCBRS, but SMARCE1-HMG and DPF2-PHD mutations are correlated to moderate-severe and mild intellectual disability phenotypes, respectively^72,74,91^. We examined non-truncating variants through predicted phenotypic severity score analysis (PolyPhen HumVar^92^), which highlighted domains such as the SMARCB1-CTD, ARID2-ARID and SMARCA2-Helicase-C and SMARCA2-post-Helicase-C as those predicted to result in most severe disease phenotypes, in agreement with published phenotypic data (Fig. 2f and Supplementary Table 3). This analysis also highlighted the SMARCC1-post-SWIRM interdomain with a particularly high PolyPhen score and average number of mutations; this region lacks 3D structural definition, implicating an alternative contribution to mSWI/SNF function (Fig. 2f). Collectively, these results highlight convergent clinical outcomes stemming from mSWI/SNF gene disruption, with variation in severity observed across distinct proteins and even domains of mSWI/SNF complex components.

### Mapping NDD missense/inframeshift variants on 3D SWI/SNF-nucleosome models

**Fig. 3:**
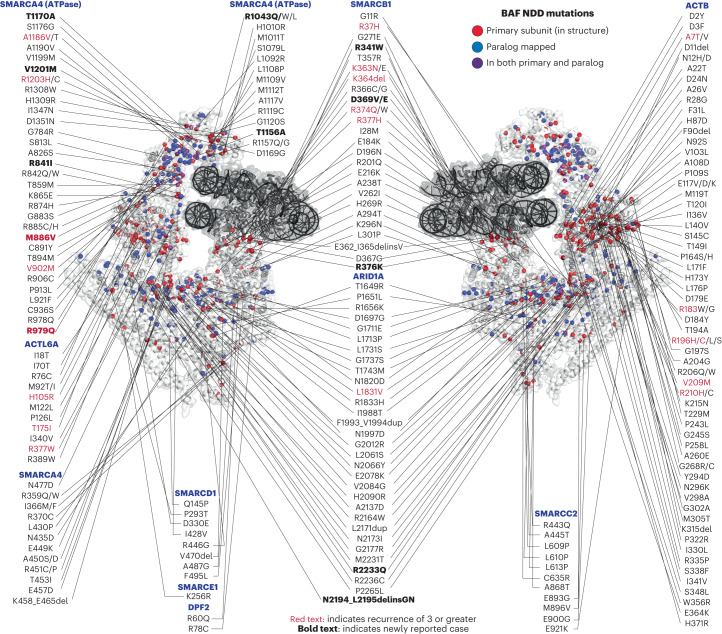
Mapping of 238 unique NDD-associated variant positions onto the structure of the human cBAF complex. NDD-associated variants, including 14 novel variants, mapped on to the 3D structure of the human cBAF complex (PDB:6LTJ). Residues shown in red spheres represent NDD-associated variants in the subunit indicated, residues in blue represent those mapped from the paralog subunit, and residues in purple represent NDD-variants mapped in both the primary subunit present on the cBAF structure and paralog mapped subunit. Recurrent variants (*n* ≥ 3) are emphasized in red text. Caution is needed when evaluating these variants in a clinical context since not all variants are confirmed as causal.

**Extended Data Fig. 3:**
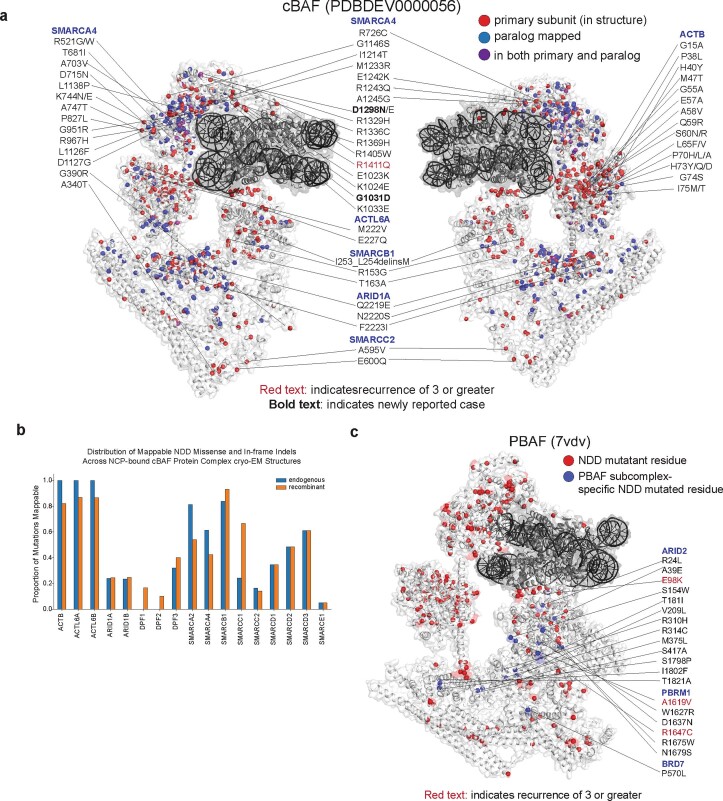
NDD-associated missense variants mapped on cBAF and PBAF 3D structures. **a**, NDD-associated missense and inframe indel variants mapped on to the 3D structure of the endogenous human cBAF complex (PDBDEV_00000056). Red spheres represent NDD-associated variants in the subunit indicated, blue spheres represent those mapped from the paralog subunit, and residues in purple represent NDD-variants mapped in both primary subunit present on cBAF structure and paralog subunit. Variants that map exclusively on endogenous complex are indicated. Recurrent variants (n>3) are emphasized in red. **b**, Bar chart indicating proportion of NDD-associated missense and in-frame indel mutations in the integrated dataset mappable to current mSWI/SNF complex structures separated by subunits. **c**, NDD-associated missense and inframe indel variants mapped on to the 3D structure of the PBAF complex (PDB 7VDV). Red and blue spheres represent NDD-associated variants in the subunit indicated. Blue spheres and annotations emphasize PBAF subcomplex specific variants mapped.

We next integrated these sequence variant data with recently solved structures of mSWI/SNF cBAF complexes^93,94^, which allowed for mapping of 238 unique positions comprising 44.08% (655/1,486) of the theoretically mappable cBAF-specific NDD missense and in-frame indels on the recombinant cBAF cryo-EM structure, and 51.55% (766/1,486) on the endogenous structure for all cBAF paralogs (Fig. 3, Extended Data Fig. 3a, b and Supplementary Table 3)^95,96^. These results highlight the need for further structural efforts as well as studies to define the roles and interactions of non-structured, disordered regions. Mapping subcomplex-specific positions onto the recently solved PBAF complex bound to a nucleosome^97^ resolved 20 additional PBAF-specific subunit mutations across ARID2, PBRM1 and BRD7 (Extended Data Fig. 3c). For ARID1B, SMARCA2 and ACTL6B, paralog subunits that are not part of the solved protein complex, we mapped mutant residues on to the respective paralogs following paralog alignment (Fig. 3 and Extended Data Fig. 3a).

**Fig. 4:**
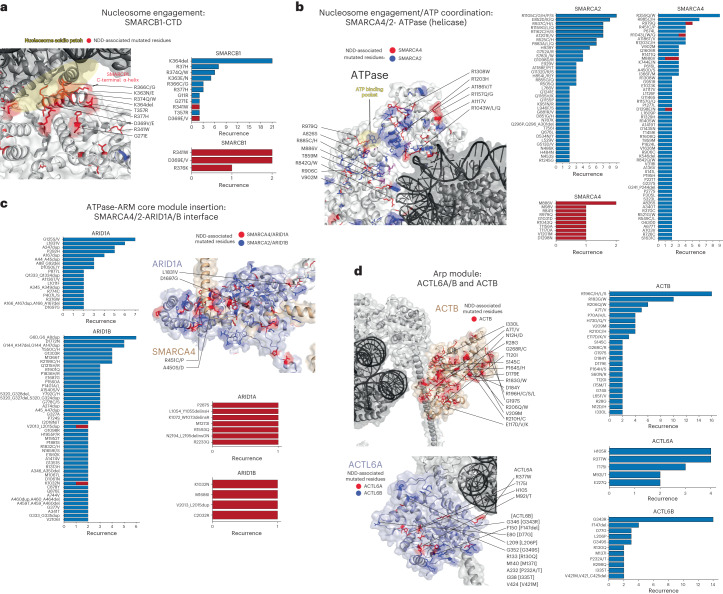
NDD-associated mutations cluster within key structural hubs of mSWI/SNF complexes. **a**, Zoomed-in view of the SMARCB1 C-terminal alpha-helix domain (PDB:6LTJ) with the nucleosome acidic patch interaction site highlighted in yellow (left). NDD-associated mutations in SMARCB1 are emphasized in red. All NDD-associated SMARCB1–C terminal alpha-helix mutations ranked by frequency (right). Novel SMARCB1 variant cases reported in this study shown in red bar chart. **b**, Zoomed-in view of the SMARCA4 ATPase subunit within the cBAF complex (PDB:6LTJ) at its interface with the nucleosome (left). Mutations in SMARCA4 are indicated in red; mutations in SMARCA2 are indicated in blue, shared mapped in purple. ATP binding pocket is highlighted in yellow. NDD-associated missense and inframeshift variants in SMARCA4 and SMARCA2, ranked by frequency, filtered for recurrence of *n* ≥ 2 by position (right). Novel SMARCA4 cases reported in this study shown in red bar chart. **c**, NDD-associated mutations in ARID1A and ARID1B, ranked by frequency, filtered for recurrence of *n* ≥ 2 by position (left). Zoomed-in view of the SMARCA4-ARID1A interface within the core module of the cBAF complex (right). SMARCA4 is shown in tan and ARID1A in light purple, with mutations in SMARCA4 and ARID1A shown in red and those in their respective paralogs SMARCA2 and ARID1B shown in blue. Novel ARID1A/B variant cases reported in this study shown in red bar chart. **d**, Left, zoomed-in view of the ACTB (tan) and ACTL6A (light purple) subunits within the Arp module of the cBAF complex, with mutations indicated in red and blue for ACTL6A paralog subunit, ACTL6B. NDD-associated mutations in ACTL6A, ACTL6B and Actin, ranked by frequency, filtered for recurrence of *n* ≥ 2 by position (right). Recurrent ACTL6B variants donated in brackets mapped onto ACTL6A indicated.

**Extended Data Fig. 4:**
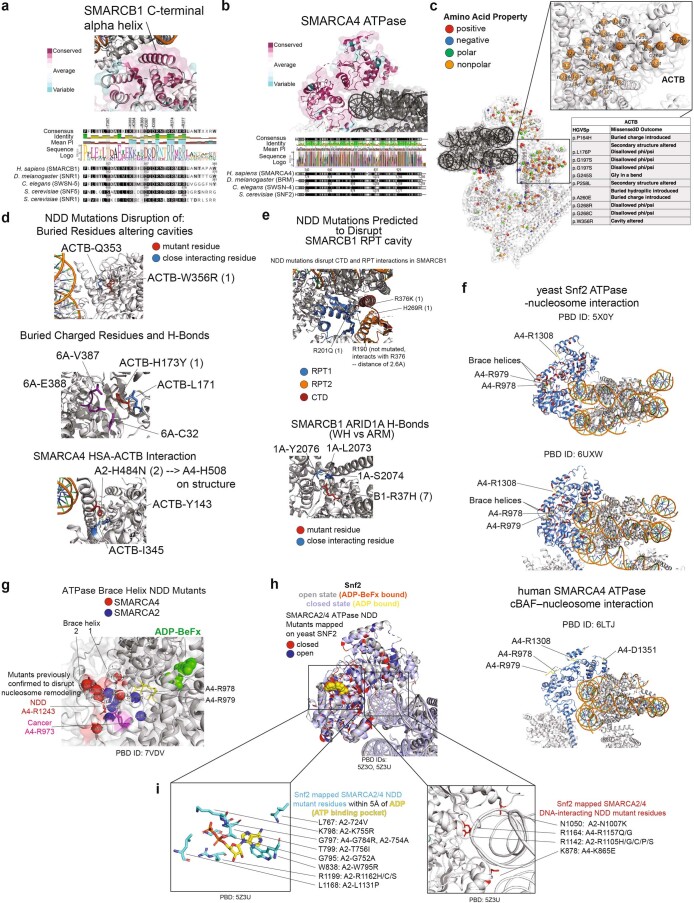
Structural dissection of mSWI/SNF subunit mutations across the ARP, Core, and ATPase modules. **a, b**, (a) SMARCB1-C terminal alpha helix and (b) SMARCA4-ATPase domain (top) ConSurf conservation mapping and (bottom) multiple sequence alignment using D. melanogaster, C. elegans, and S. cerevisiae homologs. **c**, NDD-associated missense and in-frame indel variants mapped onto the 3D structure of the cBAF complex (PDB:6LTJ) color coded by residue chemical characteristics: red: positive charge, blue: negative charge, green: polar, orange: nonpolar. Nonpolar residues of the ACTB (Arp module) and Table of nonpolar mutations predicted to structurally disrupt ACTB are shown. **d**, ACTB NDD mutations may alter internal hydrophobic cavities, interfaces with ACTL6A/B, and interfaces with SMARCA2/A4-HSA. Mutant residues shown in red and putative proximal/interacting residues shown in blue/purple. **e**, SMARCB1-RPT and WH domain NDD mutations predicted to disrupt internal cavity integrity, and hydrogen bonding to interacting ARID1A main chain carbonyls, respectively. Top, selected NDD-associated SMARCB1 missense mutations are labeled, and major domains of SMARCB1 are colored, including RPT1 (blue), RPT2 (orange), and CTD (red). Bottom, mutant residue shown in red and putative proximal/interacting residues shown in blue. **f**, Mapping of conserved SMARCA2/4 NDD mutant residues (red) on the yeast Snf2 ATPase domain (5X0Y and 6UXW) compared to the recombinant cBAF SMARCA4 ATPase (6LTJ). Brace helices (indicated in yeast structures) are not resolved in human cBAF structure, but demonstrate that certain residues, emphasized in yellow, are buried by the SMARCA2/4 brace helices, rather than exposed. **g**, Mapping of SMARCA2/4 brace helix NDD variants onto the closed state of the SMARCA4 ATPase domain using the PBAF structure (7VDV). NDD variants clustered in brace helices are predicted to disrupt nucleosome remodeling activity as has been shown with R1243 and R973 NDD and cancer-associated mutations indicated in panel^97^. **h**, Mapping of SMARCA2/4 NDD mutant residues on the Snf2 ATPase open (gray) and closed (pale cyan) states (PDB IDs: 5Z3O, 5Z3U). NDD residues colored blue in open state and red in closed state. **i**, SMARCA2/4 NDD mutant residues (left) within 5Å of the ADP-BeFx and (right) interacting with nucleosomal DNA mapped onto the closed yeast Snf2 ATPase structure (5Z3U).

This structural analysis reveals that BAF complex compromises in NDD cluster primarily in four distinct regions on mSWI/SNF complexes: the catalytic ATPase module, the mSWI/SNF core, the Arp module, and the SMARCB1 BAF-nucleosome contact point (Fig. 4a–d). As demonstrated initially through our previous work^98^ and later resolved in 3D structural efforts, CSS-associated mutations in SMARCB1 localize to the SMARCB1-CTD, the key and only interface connecting the mSWI/SNF core module to the nucleosome acidic patch (Fig. 4a and Extended Data Fig. 4a). Second, mutations in the SMARCA4 ATPase subunit are primarily situated in the ATP-coordinating and DNA-binding residues near the nucleosome, with additional mutations accumulating within the region of SMARCA4 interfacing within the mSWI/SNF core (Fig. 4b,c and Extended Data Fig. 4b). We also identified a cluster of variants are found throughout the ACTB subunit of the Arp module, whose mutation is associated with severe cases of Baraitser-Winter cerebrofrontofacial syndrome^75,95^ (Fig. 4d).

Intriguingly, whereas mutations to positively charged residues within the SMARCB1-CTD disrupt binding to the nucleosome and result in severe intellectual disability^93,94,98^, we report two novel variants in the SMARCB1-CTD, D369E and R376K, in which a positive or negative charge is maintained, and which are phenotypically associated with less severe disease (Fig. 4a, red, and Supplementary Table 1), underscoring that defining chemical properties of distinct mutations, even within a given subunit domain, may inform intellectual disability severity and phenotypic outcomes.

We next mapped cBAF NDD-mutant residues by amino acid characteristics (that is, charged, polar, nonpolar, etc). This map highlighted that many NDD-associated ACTB residues are nonpolar, the mutation of which is predicted to disrupt hydrophobic core as further suggested by Missense3D^96,99^ (Extended Data Fig. 4c and Supplementary Table 3; http://missense3d.bc.ic.ac.uk/). Within the context of mSWI/SNF (ACTB is also a member of INO80 and TIP60 complexes; Supplementary Table 2), ACTB mutations are predicted to alter buried hydrophobic cavities, as well as interaction with the ACTL6A Arp module binding partner, and even the HSA helix of the SMARCA4 ATPase (Extended Data Fig. 4d). Intriguingly, some of the most recurrent ACTL6A and ACTL6B mutations of the Arp module, R377W and G343R, are located in close proximity to one another when mapped onto ACTL6A subunit on the cBAF structure (Fig. 4d). Although not interfacing other mSWI/SNF subunits, these residues are oriented toward the DNA exit, and we hypothesize that the ACTL6A-R377 residue may stably bind the DNA backbone adjacent to the nucleosome, which would be disrupted upon mutation to a nonpolar residue such as tryptophan (R377W). Conversely, the addition of a positive charge in ACTL6B from side chain-absent glycine (G) to arginine (R) upon mutation may impart affinity to the nucleosomal DNA.

We predicted that SMARCB1 mutations in the RPT2 domain may disrupt the RPT domain cavity (Extended Data Fig. 4e). Further, the recurrent SMARCB1-R37H mutation in the winged-helix DNA-binding domain, which causes severe intellectual disability and Kleefstra-like syndrome, also demonstrated hydrogen bonding with the carbonyl backbone of ARID1A-L2073 and Y2076 that is likely disrupted upon mutation (Extended Data Fig. 4e). Intriguingly, the SMARCB1-WH domain is isolated from the SMARCB1 C-terminus on the recombinant cBAF structure but is predicted to be repositioned closer to the nucleosome binding lobe in the PBAF structure^97^, suggesting potentially distinct roles and functional impacts of the SMARCB1-R37H mutation in cBAF compared to PBAF, perhaps independent of remodeling activity as the SMARCB1-R37H mutation does not impact cBAF nucleosome remodeling activity in vitro^98^.

### Yeast SWI/SNF ATPases offer NDD variant functional insights

Given the high frequency of mutations within the catalytic ATPase subunits of mSWI/SNF chromatin remodeling complexes, SMARCA2 and SMARCA4, we mapped conserved mutant residues onto the nucleosome-bound yeast SWI/SNF and SNF2 structures^100,101^ (Extended Data Fig. 4f). Interestingly, the current human cBAF structures do not resolve the brace helices, and we highlight residues that are buried in the brace helices (SMARCA4 978-979) (Extended Data Fig. 4f). Cancer- and NDD-associated mutations (R973W and R1243W) in the brace helices of SMARCA4 were recently found to diminish nucleosome remodeling activity of PBAF complexes in vitro^97^. Given their proximity to this region and the ATP pocket of SMARCA4, we posit that additional variants in the brace helices and the nearby R978Q and R979Q variants would have similar deficits in nucleosome remodeling in human cells (Extended Data Fig. 4g). To assess the potential impact that NDD-associated mutations might have on ATP engagement, given that structures are static, we mapped conserved SMARCA2/4 mutant residues onto the open state, ADP bound (similar to apo structure) and onto the closed, ADP-BeFx-bound yeast SNF2 nucleosome bound structures^102^, which allows mapping of ~85% of all SMARCA2/4-ATPase positions (Extended Data Fig. 4h). Furthermore, this mapping highlighted NDD-associated nucleosome binding residues such as N1050 and K1057 (corresponding NDD variants: SMARCA2-N1007K and K1044E), which were previously shown to dramatically diminish nucleosome remodeling activity without disrupting ATPase consumption^102^. Mutation of additional nucleosome DNA-binding residues including K878, R1164 and R1142 (corresponding NDD variants: A4-K865E, A4-R1157Q/G, A2-R1105H/G/C/P/S) may have similar biochemical outcomes (Extended Data Fig. 4i). However, NDD-mutant residues in the ATP binding pocket are expected to disrupt the fundamental ATPase activity of SNF2. For example, mutation of either G797 or G795 (corresponding NDD variants A2-754A, A2-G752A and A4-G784R) residues, which provide space for ATP to bind to the ATP pocket, may reduce mSWI/SNF nucleosome remodeling activity (Extended Data Fig. 4i). Further work is required to define how mutations might impact the dynamic activity of these complexes as well as fully characterizing the structural domains not yet resolved in SMARCA2/4.

### Comparing cancer and NDD mutations reveals disruption hubs

**Fig. 5:**
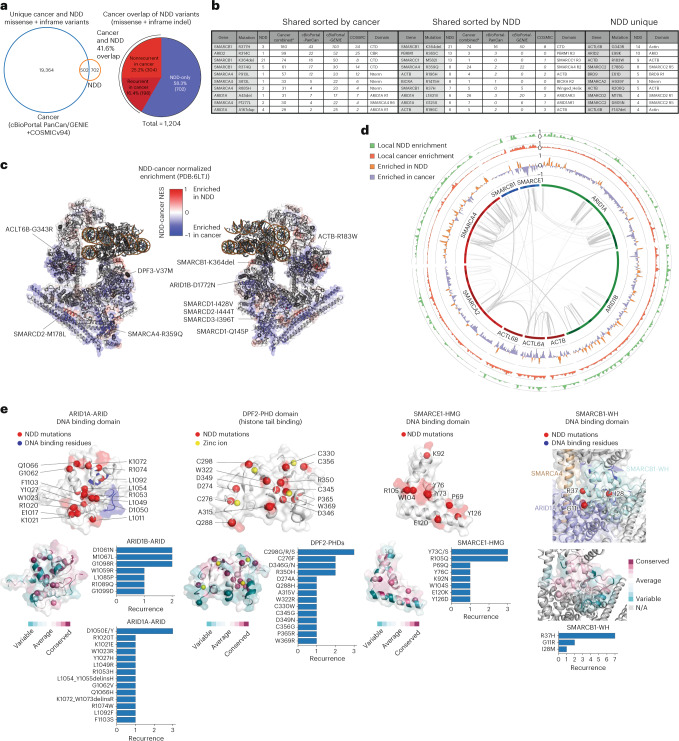
Comparison of NDD- and cancer-associated mutations in mSWI/SNF complex components. **a**, Venn diagram overlapping unique cancer and NDD missense and in-frame variants (left). Pie chart reflecting breakdown between NDD- and cancer-associated mSWI/SNF missense and in-frame mutations (right). The breakdown of recurrent and non-recurrent cancer variants is shown. **b**, Top ten recurrent missense and in-frame indel mutations specific to NDD and those shared between NDD and cancer, sorted by frequency in each disease type. Inter- and intradomains are indicated. **c**, Heatmap representation of mutation differences between NDD and cancer (NDD - Cancer normalized enrichment scores (Methods)) reflected on the 3D structure of the human cBAF complex (PDB:6LTJ). Red regions represent those enriched in NDD, blue represent those enriched in cancer (−1, maximally enriched in cancer; 1, maximally enriched in NDD). Labels for NDD hotspots are shown. **d**, Circos plot reflecting regions of top-mutated mSWI/SNF subunits and the local enrichment of missense and in-frame indel mutations in NDD (green), Cancer (red) or NDD-Cancer difference (represented as NDD-Cancer NES): NDD (orange) or cancer (purple); interactions between subunits, determined by cross-linking mass-spectrometry (CX-MS) performed on endogenous cBAF complexes are shown (NCP-bound endogenous cBAF, from Mashtalir et al.^93^). Scaled local recurrence, and NDD-Cancer NES were calculated similarly to panel c with one exception, where all secondary paralog mutations were preserved instead of remapping to paralogs. Enrichment scores were bounded from 0 to 1 for local recurrence and −1 to 1 for differential enrichment of mutations. Domains are represented as darker bands in the first inner ring of the Circos plot. **e**, NDD-associated mutant residues emphasized as red spheres on the structures of the ARID1A-ARID domain (PDB:1RYU), the DPF2-PHD domain (PDB:5B79), the SMARCE1-HMG DNA-binding domain (PDB:7CYU) and the SMARCB1-winged-helix DNA-binding domain (PDB:6LTJ). NDD-associated missense and inframeshift variants, ranked by frequency, are shown as bar charts. ConSurf conservation scores are mapped onto each domain structure with cyan-white-magenta color scale in increasing conservation order.

**Extended Data Fig. 5:**
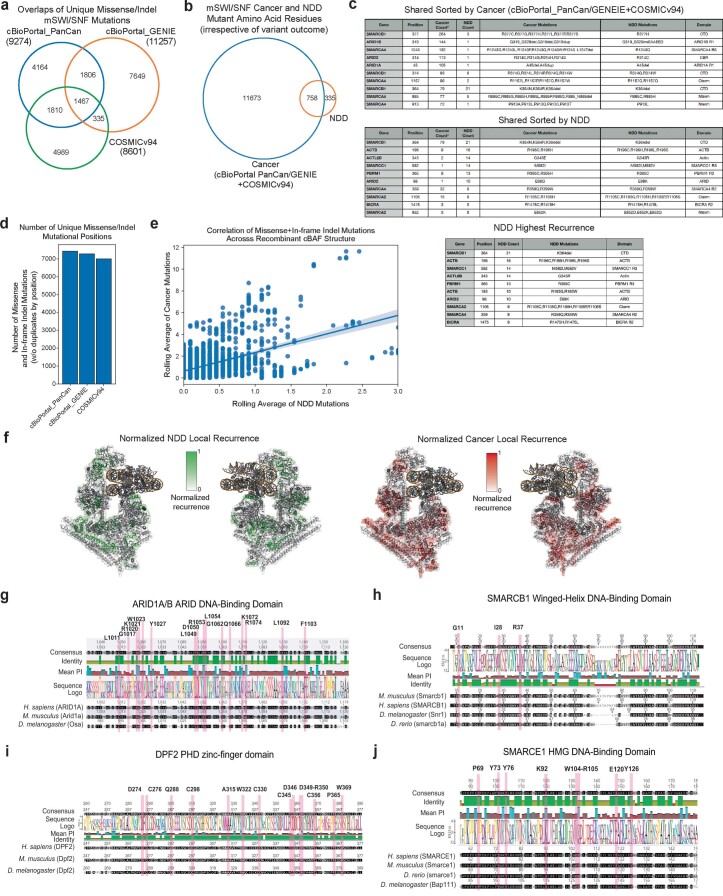
Perturbed subunit positions shared between cancer and NDD highlight ATPase, nucleosome binding regions, and Arp module. **a**, Venn diagram overlapping unique cancer missense and inframeshift variants identified from cBioPortal_PanCan, cBioPortal_GENIE and COSMICv94 cancer genetics datasets. **b**, Venn diagram overlapping unique cancer and NDD (Supplementary Table 1) missense and inframe variants by amino acid position regardless of mutation consequence. NDD mutations derived from Supplementary Table 1, cancer mutations derived by combining cBioPortal_PanCan, cBioPortal_GENIE and COSMICv94 datasets. **c**, Top ten most recurrent mutant residue amino acid positions shared between Cancer and NDD sorted by frequency in each disease type. Highest recurrence of NDD mutations also included. NDD- and cancer-associated mutations were derived as described in (b). **d**, Bar plot showing the total number of unique missense/indel mSWI/SNF mutations across the following cancer datasets: cBioPortal_PanCan, cBioPortal_GENIE, COSMICv94. **e**, Correlation of missense and inframeshift mutations shared between cancer (cBioPortal_PanCan only) and NDD across recombinant cBAF structure. Briefly, NDD- and cancer-associated missense and in-frame indel mutations were remapped onto the primary paralogs of the recombinant cBAF (PDB ID: 6LTJ) structure. A rolling average with a window size of 11aa centered on each residue (5aa on each side) of mutation recurrence by residue position for NDD and cancer was used for the scatterplot and correlation calculation. NDD- and cancer-associated mutations were derived from Supplementary Table 1 (NDD) and cBioPortal_PanCan datasets. The translucent bands around the regression line represent the 95% confidence interval estimated using a bootstrap for 100 iterations. **f**, Heatmap representation of scaled local enrichment of NDD- and cancer-associated missense and in-frame indel mutational burden of (left, in green) NDD and (right, in red) cancer reflected on the 3D structure of the human cBAF complex (PDB: 6LTJ). Local enrichment scores were computed as described in (Fig. 5e). NDD- and cancer-associated mutations were derived as described in (Fig. 5e). **g–j**, Multiple sequence alignment of (g) ARID1A-ARID domain, (h) SMARCB1-WH domain, (i) DPF2-PHD domain, and (j) SMARCE1-HMG domain, with variety of related homologs (including M. musculis, D. rerio, D. melanogaster, C. elegans, and S. cerevisiae, where possible).

Previous studies have examined the distribution of cancer-associated single-residue mutations on the cBAF complex structure^93,94,97^. For our analysis, we examined the overlap of unique missense and inframeshift mutations identified in the context of NDD with those in human cancer (cBioPortal-PanCancer^103,104^, AACR Project GENIE^105^ and COSMIC^86^) (Extended Data Fig. 5a). We found that the majority (58.3%) of unique mutations found in NDD were specific to NDD (Fig. 5a, Supplementary Table 4). Further, among the 41.6% of shared cancer mutations, 16.4% were found to be recurrent among the three cancer datasets analyzed (Fig. 5a). Shared recurrent mutations in both NDD and cancer included those localized to the C-terminal domain of SMARCB1, the SMARCA4 N terminus, as well as within PBRM1 and ACTB subunits (Fig. 5b and Supplementary Table 4). By examining mutational positions rather than unique mutations, we found that over two thirds (69.3%) of NDD-mutant positions are also altered in cancer, with similar breakdown of the shared mutational recurrence (Extended Data Fig. 5b,c). Given the difficulty of de-duplicating cancer variants across the three cancer databases used in this study (cBioPortal PanCan/GENIE and COSMIC datasets), we used the cumulative recurrence across the three datasets for comparison to NDD recurrence (Fig. 5b, Extended Data Fig. 5c,d and Supplementary Table 4).

A minor positive correlation was observed between the recurrence of shared cancer (cBioPortal-PanCan) and NDD sequence variants (Extended Data Fig. 5e). Although normalization of both NDD and cancer mutational frequencies can mask regions highly mutated in both disease settings, mutational enrichment analyses revealed several unique mutational hot spots specific to human NDD (Fig. 5c). Mutations in Arp module subunits, ACTB and ACTL6A/B, were nearly selectively enriched in NDDs, whereas mutations in the helicase domain of SMARCA4 were more enriched in cancer (Fig. 5b,c). Mutations overlapping with those in cancer localize to the SMARCA4 ATP binding pocket and nucleosomal DNA-binding residues, the SMARCB1-CTD, and the SMARCA4-BAF core module entry point (Fig. 5b and Extended Data Fig. 5f). Finally, we used cross-linking mass spectrometry (CX-MS) datasets from previous studies performed on endogenous cBAF complexes^16^, which further demonstrated region-specific enrichment of NDD-versus cancer-associated mutations throughout cBAF subunits (Fig. 5d).

### Mutations in structurally and functionally elusive domains

To date, 3D structural studies have resolved only ~44% of the total cBAF complex (by molecular weight), owing to the presence of low-complexity or disordered regions within many subunits (with to-date unassigned functions). Further, and given that such regions are often spaced between structured domains, several structured domains, many solved in isolation, have not been solved in the context of full 3D cBAF or PBAF complexes. We thus mapped all NDD non-truncating variants to the highly mutated ARID1A-ARID DNA-binding domain, the SMARCE1-HMG domain, the DPF2-PHD domains and the SMARCB1-WH domain to previously resolved high-resolution apo structures^106–109^ (Fig. 5e and Extended Data Fig. 5g–j). Intriguingly, the majority of ARID1A-ARID domain and SMARCB1 WH domain non-truncating variants do not overlap with the DNA-binding residues, and we therefore predict that they disrupt intradomain structural integrity (Fig. 5e and Extended Data Fig. 5g–h)^108^. As has been demonstrated previously, mutations in the DPF2-PHD domains disrupt zinc-binding residues which are important for PHD domain structural formation, resulting in decreased affinity to modified histone substrates (Fig. 5e and Extended Data Fig. 5i)^109^. NDD-associated mutations in the SMARCE1-HMG domain accumulate on the DNA-binding interface of the structure (Fig. 5e and Extended Data Fig. 5j)^107^ and hence are predicted to inhibit DNA binding.

## Discussion

**Fig. 6:**
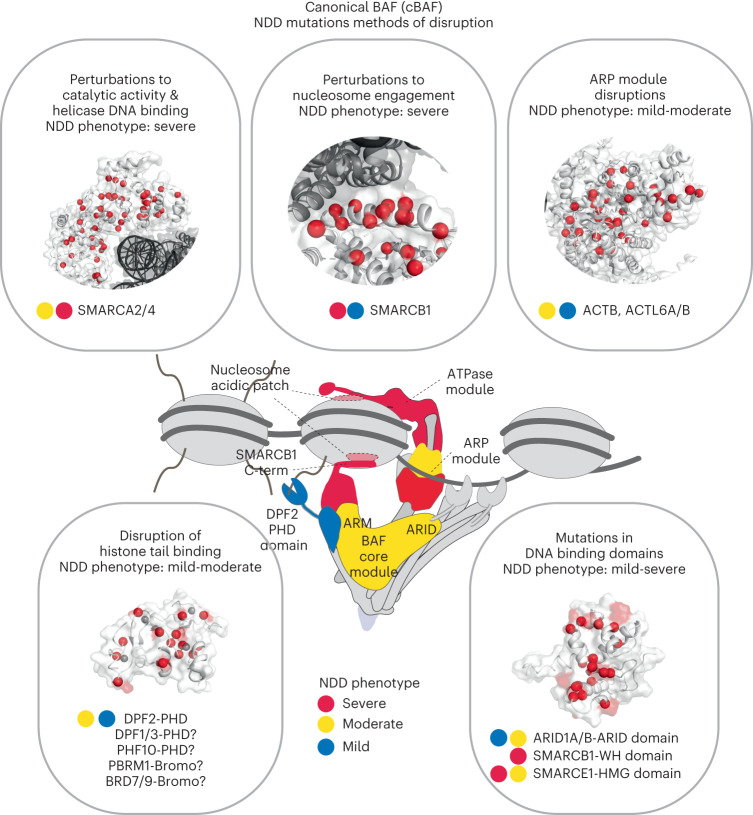
Summary of widely disrupted mSWI/SNF complex hubs in NDDs. NDD-associated mSWI/SNF mutations occur across several subunits of the mSWI/SNF family of chromatin remodeling complexes and cluster in key structural hubs. Missense and in-frame deletions accumulate within the catalytic ATPase, nucleosome interacting, histone-binding or DNA-binding domains, as well as the ARP module, underscoring their convergence in producing neurodevelopmental aberrations. Interpretation of NDD-associated variants in the context of this framework enables mechanistic dissection of mSWI/SNF activities and provides functional links relevant to clinical phenotypes.

Here, we demonstrate that mSWI/SNF complex genes are the most frequently disrupted chromatin regulatory entity in NDD, with perturbation of several key structural ‘hubs’ within this multicomponent complex displaying a phenotypic convergence that yields NDD features associated in the literature with the greatest level of NDD severity (Fig. 1d and Fig. 6). Our study serves as a powerful foundation upon which to pursue integrated efforts between the chromatin biology and neurobiology communities to functionally characterize and prioritize these frequent disruptions.

It should be noted that because the products of mSWI/SNF complex genes are assembled into a highly heterogeneous group of complexes, the total extent of mutational burden of this complex reported here may not be completely recognized, even with genes such as *ARID1B* ranking among the most highly mutated in NDDs (Extended Data Fig. 1n)^110–112^. Disruption of both structured and unstructured domains presented here may impart altered mSWI/SNF complex localization and activity on the genome via a range of mechanisms requiring extensive further investigation. Additionally, further examination of zygosity and how missense variants within the same protein differentially impact protein activity may reveal distinct functions. For example, both dominant and recessive single amino acid variants affected *ACTL6B* have been identified^113^. Although the ACTL6B G393R recessive variant has been shown to reduce ACTL6B protein expression, behaving as a loss-of-function mutation^114^, the dominant G343R variant is predicted to impart dominant-negative effects that disrupting mSWI/SNF activity^42^.

In this study, we curated a list of chromatin regulatory genes in combination with the EpiFactor database to investigate the prevalence of chromatin-related process disruptions in NDD. However, additional work is needed to define a maximally complete set of chromatin regulators, regulatory complexes and their subunit membership. Further, functional studies must be performed to define mechanisms by which variants alter activity or other functions, especially given that 3D structures are based on a range of complex states and conformations, which may vary in biologic relevance. Importantly, although we have obtained information on recurrence of sequence variants for which distinct cases were clear, potential duplicates were omitted in processing in cases for which we could not verify distinct cases between literature and databases used, meaning that recurrence of some variants may be artificially reduced. Further, cross-referencing of additional private databases such as FoundationCORE may be useful in follow-up analyses^115^. To prevent inclusion of false positives, we omitted NDD-associated mSWI/SNF sequence variants which are also present in gnomAD with a minor allele frequency of >0.5%, predicted to be benign. Although the overwhelming majority (96%) of DECIPHER variants reported to date are heterozygous (Extended Data Fig. 1l), zygosity data were not included in this study, and this remains a limitation. By centering the majority of our analysis on de novo variants, we expect these to be pathogenic; however, future studies must be performed to assess the full scope of the molecular and pathophysiological consequences of these mutations.

## Methods

### Novel variant collection

Novel NDD-related mSWI/SNF gene variants reported in this study were identified through physician referrals and the Coffin-Siris syndrome registry. Variants from Leiden University Medical Center were identified in a diagnostic setting, and genetic data were retrieved from the generated reports or shared with us by the treating physician with consent from the patient or parents. The institutional review board of Leiden University Medical Center provided approval waivers for using de-identified data and publishing aggregated data (G18.098 and G21.129) without obtaining specific informed consent. Individuals identified through Eastern Virginia Medical School were recruited to the Coffin-Siris syndrome registry through clinicians, social media and patient foundations. Individuals completed an online consent form followed by a registry survey with phenotypic questions. The Coffin-Siris Syndrome Registry has been approved by the Eastern Virginia Medical School institutional review board (15-03-EX-0058). Novel variants reported in this study have been deposited in LOVD (https://www.lovd.nl/)^24^. Variants identified through this method that were present in previously published literature or deposited in an online repository were excluded for analysis in this study to prevent reporting potential duplicates (Curating mSWI/SNF gene NDD-associated variants section). Given that our paper centers on the mutational rather than phenotypic outcomes of NDD-related mSWI/SNF variants, future clinical papers will further explore the phenotypes associated with novel variants published in this manuscript. During the review process, some novel variants included in this study were published with detailed clinical information^89^.

### Mutational datasets

Open-access mutations publicly available on the DECIPHER database (https://www.deciphergenomics.org/; accessed June 22, 2022) (ref. ^22^) were used for broader chromatin gene analysis (Fig. 1c,d and Extended Data Fig. 1k,l). The queried chromatin remodeling complex gene list (SWI/SNF, CHD, INO80 and ISWI) was manually curated from a literature review detailed below (Supplementary Table 2).

### Chromatin regulatory gene sets (Supplementary Table 2)

Chromatin remodeling complex gene lists were curated from a variety of sources, including HGNC gene groups SWI/SNF and INO80 (https://www.genenames.org/data/genegroup/#!/), as well as a literature review of all chromatin remodeling complexes^116,117^, mSWI/SNF^16^, ISWI^118^, CHD^119^ and INO80 (refs. ^120–124^). The histone modifier gene list was gathered from *HISTome2* (refs. ^125,126^) (http://www.actrec.gov.in/histome2/). Polycomb repressive complex genes and DNA methylation regulatory genes were informed by the literature^127,128^. Additional chromatin regulatory complexes were obtained from EpiFactor^82^ (https://epifactors.autosome.org/protein_complexes). The full set of cBAF, PBAF and ncBAF genes were included in the EpiFactor complexes if absent.

### Curating mSWI/SNF gene NDD-associated variants

The set of rare inherited and de novo variants included data from three cohorts of individuals with autism spectrum disorders or other developmental disorders: the Simons SSC/ASC, SPARK and DDD cohorts. Details about merging and de-duplicating the data are described in Fu et al.^129^. Briefly, duplicated samples were identified and excluded by IBD and other metadata, and the filtered samples were merged to provide a single unified set of de-duplicated de novo variants in autism spectrum disorders and other developmental disorders. The recurrence of NDD de novo variants across BAF genes and several gene sets of interest, including a curated set of chromatin remodelers, epigenetic modifiers and synaptic genes were visualized with scatter plots and bar charts using matplotlib^130^. The set of de novo variants and non-benign SNVs in DECIPHER were used for all summary calculations in Fig. 1 and Extended Data Fig. 1 and for comparisons between the BAF genes, chromatin regulatory genes, epigenetic modifier genes and synaptic genes. The queried chromatin regulatory gene list was based on EpiFactor (https://epifactors.autosome.ru/genes; accessed 2 September 2021) (ref. ^82^ updated to include all mSWI/SNF genes (Supplementary Table 2). The queried synaptic gene list was based on the SynGO gene list (https://www.syngoportal.org/; accessed 2 September 2021) (ref. ^83^). The development disorder DECIPHER gene list was based on DDG2P genes in DECIPHER (accessed 13 June 2022).

A comprehensive list of SNV and short in-frame indels (inframeshift variants) was compiled from an extensive literature review, the combined set of rare inherited and de novo variants from the Simons SSC/ASC, SPARK, and DDD cohorts (the ‘combined cohort study’), the DECIPHER database of SNVs (https://www.deciphergenomics.org/), the merged set of de novo mutations from the DNM effort by McRae et al.^34^ NDD-associated ClinVar mutations (accessed 5/15/2021), NDD-associated variants from LOVD (LOVD v3.0 accessed June 2022) and 85 previously unreported cases published in this study collected through the laboratories of S.A.S.V. (Eastern Virginia Medical School) and G.W.E.S. (Leiden University Medical Center).

First, the combined set of rare inherited and de novo variants was split into a set of rare inherited variants and a set of de novo variants. All rare inherited PTVs, in-frame indel variants and de novo variants were included in the integrated dataset. Guided by the analysis in Fu et al.^129^, where missense variants with MPC scores (missense badness, PolyPhen-2 and constraint) of 1 or more were observed to confer moderate to strong levels of risk in developing autism and missense rare inherited variants with MPC scores ≥1 were included in the integrated dataset. All other rare inherited variants from the combined cohort study were excluded. Then, samples were cross-referenced between the combined cohort study, DECIPHER database, and the DNM cohort of de novo mutations and identical variants from the same samples (using available sample IDs or aliases) were removed to de-deduplicate the data between these three cohorts/databases. Separately, a list of de novo variants in BAF genes across several other studies in the literature not covered previously by the cohorts used in DECIPHER and the combined cohort study (SSC/ASC, SPARK and DDD) were manually curated and de-duplicated to form the compiled set of mutations from the literature. Additionally, NDD-associated mutations from the LOVD database were compiled and filtered to include all PTV and in-frame indels and de novo/likely de novo missense variants. All benign/likely benign variants were excluded. The filtered set of LOVD variants and the manually curated variants from the literature were merged and de-duplicated based on sample IDs or aliases (if available) and study ID / reference (if sample IDs were not available). For shared variants between LOVD and the literature, where it was not clear whether these variants were duplicates, only shared variants from the manually curated literature dataset were kept, effectively de-duplicating the data. Minimal overlap was assumed between the de-duplicated set of LOVD/literature variants and the de-duplicated set of SSC + ASC/SPARK/DDD/DECIPHER/DNM variants. These two sets were merged, followed by a round of manual curation to double check that as many duplicates or potential duplicates were removed during dataset integration. The set of 85 novel cases identified by S.A.S.V. and G.W.E.S. were added to this merged dataset. In parallel, a curated set of ClinVar variants from samples with NDD-associated clinical features and unknown/likely pathogenic/pathogenic clinical significance was generated. Benign and likely benign ClinVar variants were excluded. Additionally, ClinVar variants submitted by GeneDx were excluded due to substantial overlap with the comprehensive analysis of de novo mutations in NDD by Kaplanis et al. included in the DNM database of de novo mutations. Samples were de-duplicated between ClinVar and the LOVD/literature dataset using SCV codes wherever available. Finally, this de-duplicated ClinVar dataset was used to adjust the counts of the previously merged dataset of NDD-associated BAF mutations from the combined cohort study (SSC/ASC, SPARK and DDD), DECIPHER SNVs, DNM, LOVD and the literature. It was difficult (and sometimes impossible) to track, match and assign each filtered NDD-associated ClinVar SCV (submitted record for each variant) with the list of available sample IDs or aliases in the previously merged dataset. Thus, the total counts for each variant were adjusted to the total counts found in ClinVar (based on the number of submissions for each variant using SCV IDs) to eliminate the possibility of double counting if the ClinVar total count for a variant was more than the total count from the previously merged dataset. This procedure assumes submissions to ClinVar overlap entirely with the previously merged dataset, so it is possible the new merged dataset containing ClinVar variants might undercount some NDD-associated BAF variants. This integrated dataset was compared to gnomAD v3.1.2 to remove potential SNPs and other variants that occur frequently in a collection of healthy individuals. A more stringent MAF threshold of ≥0.5% MAF was used to exclude potentially common variants in the integrated dataset. This final integrated dataset was manually checked once more to exclude potential duplicates and likely benign variants before freezing for all downstream analyses. A total of 2,539 NDD-associated BAF variants are included in this dataset, including 85 novel cases and 72 previously unreported variants.

To standardize the data, all variants were remapped to the UniProt canonical BAF protein isoforms (see Supplementary Table 3), and duplicates that could not be confirmed unique cases were removed. Unless otherwise noted, remapping of all variants (both NDD variants and cancer variants) to different isoforms was performed using the Ensembl Variant Effect Predictor (VEP) online web server^131^.

gnomAD variants of the general population were derived from the gnomAD v3 dataset (accessed 11 January 2021).

### Cancer dataset cleaning and compilation

PanCancer datasets from TCGA and cBioPortal^103,104^ were cleaned and compiled for all downstream analyses related to NDD versus cancer comparisons.

The TCGA MC3 PanCancer dataset was used for NDD versus cancer comparisons in Extended Data Fig. 1. Briefly, known SNPs were removed and BAF gene mutations were remapped to the canonical UniProt transcripts (Supplementary Table 3). Missense, nonsense and frameshift mutations were included, and all other mutations were excluded. This filtered set of mutations merged with the combined cohort study of NDD-associated mutations from the combined SSC/ASC, SPARK and DDD cohorts. Total cancer missense, frameshift and nonsense mutational recurrence was log normalized, compared to total de novo NDD-associated missense and PTV mutational recurrence for each gene, and visualized as a scatterplot using matplotlib^130^, with BAF genes indicated in red. The total proportion of NDD and Cancer missense and PTV mutations across the BAF genes were visualized as a grouped bar chart using matplotlib^130^.

Mutations across BAF genes from the curated set of nonredundant studies in cBioPortal, the AACR Project GENIE (accessed through cBioPortal) and COSMIC were compiled and filtered for NDD versus cancer comparative analyses across the BAF genes. Briefly, the BAF mutations were remapped to the UniProt canonical BAF protein isoforms (Supplementary Table 3) using the Ensembl VEP online web server^131^. Missense, frameshift, nonsense and in-frame indels were included, and all other mutations were excluded. Additionally, duplicate mutations in patients with multiple samples were excluded. This filtered set of mutations from cBioPortal^103,104^ was used for downstream BAF cancer versus NDD comparative analyses.

### NDD gene set enrichment analysis

A custom Perl^132^ script was used to determine the enrichment of GOMF gene sets enriched in DDG2P genes, a list of genes known to be associated with developmental disorders. All BAF genes were added back to DDG2P gene list if absent. Specifically, GOMF gene sets were overlapped with DDG2P using gene symbols and a hypergeometric distribution test (for example, statistical overrepresentation test) was used to evaluate the significance (*P* value) of enrichment of each GOMF. Additionally, the total and mean number of de novo missense and PTVs in ASD + DD using the combined cohort study was calculated for the overlapping genes (using gene symbols) between each GOMF gene set and DDG2P genes. The enrichment of GOMFs in DDG2P genes were visualized as scatterplots and ranked by significance (*P* value) and total de novo missense and PTV mutational recurrence for the overlapping genes (using gene symbols) with the top 10 GOMFs labeled. Additionally, the top 50 most enriched GOMFs by statistical significance (*P* value) were ranked by the mean number of de novo missense and PTVs in the overlapping genes (using gene symbols) in the combined cohort study and the mean number of non-benign DECIPHER SNVs in the overlapping genes (using gene symbols) and visualized as scatter plots with the top 25 GOMFs indicated.

Further, the top 50 most enriched GOMFs by significance (*P* value) were categorized into five major groups and colored accordingly in the scatter plots. Additionally, the total number of non-benign DECIPHER SNVs for the overlapping genes (using gene symbols) in these five major groups and chromatin remodeling complexes from the curated list of chromatin regulators were visualized as a bar chart (GOMF chromatin gene sets and chromatin regulatory complexes were merged into one group).

The GOMFs gene sets were obtained from MSigDB v7.5.1 (GOMF v7.5.1; https://www.gsea-msigdb.org/gsea/msigdb/). The ARID2, BCL7A/C and BICRAL BAF genes were added to the chromatin binding GOMF gene set.

Benign and likely benign SNVs in DECIPHER were excluded to create the set of non-benign DECIPHER SNVs. The development disorder DECIPHER gene list was based on DDG2P genes on DECIPHER (accessed on 15 May 22).

### NDD recurrence in chromatin regulatory complexes, epigenetic modifiers and synaptic genes

Queried chromatin remodeling gene lists (Supplementary Table 2) were used for all downstream analysis in Fig. 1/Extended Data Fig. 1.

The total number of de novo missense and PTVs in the combined cohorts ASD + DD study (SSC/ASC, SPARK, and DDD) across a curated list of chromatin regulators and EpiFactor complexes were visualized as bar charts. The total number of de novo missense and PTVs in DD (DDD) and ASD (SSC/ASC and SPARK) across EpiFactor complexes were visualized separately as bar charts. The total number of de novo missense and PTVs in ASD + DD for every gene was visualized as a scatter plot with BAF genes indicated in red. The mean number of de novo missense and PTVs in ASD + DD (SSC/ASC, SPARK, and DDD) across EpiFactor complexes were visualized as a bar chart. Protein lengths were obtained from the top reviewed UniProtKB accession for each gene. The total de novo missense and PTVs in ASD + DD (SSC/ASC, SPARK, and DDD) for each EpiFactor complex was divided by the total protein length of each EpiFactor complex to obtain protein length-normalized NDD de novo mutational recurrence (that is average number of de novo missense and PTVs per residue in each EpiFactor complex). The protein length-normalized de novo mutational recurrence for EpiFactor complexes were visualized as a bar chart.

Benign and likely benign SNVs in DECIPHER were excluded to create the set of non-benign DECIPHER SNVs. The mean number of non-benign DECIPHER SNVs and de novo missense and PTVs in ASD + DD across all EpiFactor complex genes, mSWI/SNF genes and SynGO synaptic genes were visualized as bar charts. The total number of non-benign DECIPHER SNVs across a curated list of chromatin regulators were visualized as a bar chart.

All bar charts were created using matplotlib^130^, and mSWI/SNF and cBAF, PBAF and ncBAF gene sets are indicated in red. Ensembl gene IDs (ENSG IDs) were used to overlap genes, merge datasets, and calculate the total or mean number of de novo missense and PTVs in ASD + DD and non-benign DECIPHER SNVs for gene sets in the list of curated chromatin regulators and EpiFactor complexes (Supplementary Table 2).

### Structure figures

The mapping of unique SNV and short in-frame insertion/deletion mutations was visualized using PyMol (v2.4.0) (ref. ^133^). The structural models used for this study were the following: Recombinant cBAF structure bound to nucleosome (PDB: 6LTJ), Endogenous cBAF structure bound to nucleosome (PDBDEV: PDBDEV_00000056), PBAF complex bound to nucleosome (7VDV), SNF2h (5X0Y), yeast SWI/SNF (6UXW), ARID1A-ARID (1RYU), DPF2-PHD (5B79), SMARCE1-HMG (7CYU) and SMARCB1-WH (6LTJ). Domain annotations were obtained from the PFAM and the literature, and manually curated (Supplementary Table 3).

### Conservation analysis

Conservation analysis was performed for the recombinant cBAF structure (PDB:6LTJ; SMARCA4, chain I and SMARCB1, chain M), and the ARID1A-ARID (1RYU), DPF2-PHD (PDB: 5B79), and SMARCE-HMG (PDB: 7CYU) domains using the ConSurf Server (https://consurf.tau.ac.il/)^134^. Briefly, Protein Data Bank (PDB) IDs were selected and run through ConSurf analysis using standard parameters (HMMR search algorithm, UNIREF-90 protein database, automatic homolog selection and MAFFT multiple sequence alignment method). Once completed, amended PDB files color coded by conservation were downloaded and instructions to ‘create high resolution figures’ were followed as instructed by the ConSurf server.

### Pairwise alignment

Multiple sequence alignments of the SMARCA4-ATPase, SMARCB1-CTD, ARID1A-ARID, SMARCB1-WH, DPF2-PHD and SMARCE1-HMG domains with their respective homologous proteins were performed using Geneious Prime (v2021.2.2) using standard parameters.

### General

Unless otherwise noted, mutational counts, bar plots, heatmaps and pie charts throughout were made using a combination of R (v4.1.1), GraphPad Prism (v9.2.0) and matplotlib (v3.3.1), and seaborn.

### ConSurf mutational analysis

Full-length FASTA sequences of the UniProt canonical transcript for all mSWI/SNF genes were uploaded to the ConSurf server with default parameters to obtain predicted conservation scores. The number of missense and in-frame indel NDD mutations by gene and position and the predicted ConSurf conservation score (negative-transformed so that higher scores indicate more conserved residues) were visualized as a scatter plot. All mSWI/SNF genes were used for this analysis.

### NDD domain mutation analysis

The proportion of NDD mutations from the compiled list (missense, in-frame indels, frameshift and nonsense mutations) were summed for each gene, domain and inter-domain regions (Supplementary Table 3). The proportion of NDD mutations within domains (intradomain) and between domains (interdomain) were visualized as a stacked bar plot. Domains were defined by PFAM, UniProtKB, manual curation and resolved structures.

### NDD disorder analysis

The proportion of NDD mutations from the compiled list (missense, in-frame indels, frameshift and nonsense mutations) falling within disordered (defined by MobiDB-lite; Supplementary Table 3) and structured regions were visualized as a stacked bar chart for individual BAF genes and BAF genes as a whole collection.

### PolyPhen mutational analysis

The PolyPhen HumVar^92^ model was used to predict the severity of each missense mutation in the list of compiled NDD mutations. The number of NDD missense mutations for each intradomain (within-domain) and interdomain (between-domain) region was divided by the lengths of these regions to calculate the average number of NDD missense mutations per residue for each interdomain or intradomain region. The PolyPhen HumVar predicted severity scores for each residue in each interdomain and intradomain were summed and divided by the length of each region to calculate the average PolyPhen HumVar predicted severity score for each inter-domain and intra-domain region. The average predicted PolyPhen HumVar predicted severity score and average number of NDD missense mutations were visualized as a scatter plot with interdomain and intradomain status indicated by color. All BAF genes were used for this analysis.

### 2D schematics

The distribution of gnomAD (v3) missense SNPs were visualized as a kernel density estimate plot using the seaborn kdeplot with default parameters. The gnomAD (v3) missense mutations for SMARCA2, SMARCA4, ARID1A, ARID1B, SMARCB1, SMARCE1 and DPF2 were used to compute the missense recurrence by position across the length of each protein, which was used as input into the kernel density estimate analysis. The NDD compiled list of mutations (missense, in-frame indels, frameshift and nonsense mutations) for the aforementioned genes were visualized using the St. Jude PeCan Protein Paint software with default settings (https://proteinpaint.stjude.org/). Special care was taken to map the mutations on the canonical UniProt isoform (Supplementary Table 3). Domains using the annotations compiled from PFAM, InterPro and the literature, and manually curated based on the AlphaFold EMBL-EBI structural predictions. ConSurf conservation scores were visualized as horizontal bars using the ConSurf provided ‘COLOR’ column with an aggregation of scores (1, 2 or 3, cyan; 4, 5 or 6, white; 7, 8 or 9, violet). The coverage of the two available recombinant (PDB:6LTJ) and endogenous nucleosome-bound cBAF structures were visualized as horizontal bars (recombinant coverage in orange, endogenous coverage in red and dual coverage in brown).

### Missense DNA and protein changes

The frequencies of DNA point substitutions (all SNVs) and protein amino acid substitutions (top 20) in the compiled NDD mutation dataset (missense only) were visualized as bar plots. Additionally, the amino acid substitutions for the missense subset of mutations in the compiled NDD mutation dataset was visualized as Sankey Diagram using Google Charts. Additionally, these amino acid substitutions were aggregated into functional changes (negative, positive, polar, nonpolar and miscellaneous) and visualized as proportions in stacked bar charts.

### Mappability of NDD mutations

The proportion of NDD mutations in the compiled NDD mutation dataset (missense, in-frame indels, frameshift and nonsense mutations) mappable across the endogenous and recombinant (PDB:6LTJ) were visualized as a group bar plot (Supplementary Table 3).

### NDD versus cancer overlap analysis

The recurrence of every unique gene-mutation combination for missense and in-frame indel mutations from the NDD compiled dataset and the cBioPortal (accessed June 2022) cancer dataset was computed and visualized as a pie chart or tables.

### NDD versus cancer NESs and comparative analyses

The missense and in-frame indel mutations from the compiled NDD mutation dataset and the cBioPortal cancer dataset were used to compute the NDD and cancer mutation recurrence by position across each BAF gene. This recurrence was scaled between 0 and 1 using the MinMaxScaler preprocessing function in scikit-learn. The rescaled mutation recurrence for cancer was subtracted from the rescaled mutation recurrence for NDD to compute the NDD-Cancer normalized enrichment scores (NESs). Specifically, cancer NESs were calculated using a four-step process. First, paralogs were pairwise aligned to the primary paralog, and mutations on conserved residues were remapped from the secondary to the primary paralogs. Second, the mutational recurrence by residue position of NDD- and cancer-associated missense and in-frame indel mutations were calculated across all mSWI/SNF subunits and averaged over a window size of 21 aa centered at each residue (10 amino acids on each side). Third, these smoothed averages were scaled to a range between 0 (no recurrence) and 1 (highest recurrence) to generate the local recurrence of NDD- and cancer-associated missense and in-frame indel mutations. Fourth, the local recurrence maps across all mSWI/SNF for NDD- and cancer-associated mutations were subtracted (NDD-cancer) to form the NDD-cancer NES on a range bounded by −1 (maximally enriched in cancer) and 1 (maximally enriched in NDD). NDD- and cancer-associated missense and in-frame mutations were derived as described in (Fig. 5a). These local and NESs were visualized across the specific paralogs in the recombinant cBAF structure (PDB ID 6LTJ) as various colored heatmaps (local NDD recurrence scaled in green, local cancer recurrence scaled in red, NDD-Cancer NESs in blue-white-red: blue = enriched in cancer, red = enriched in NDD) and across specific paralogs indicated in the Circos plot as a purple-orange histogram (purple, enriched in cancer; orange, enriched in NDD). The local enrichment scores for NDD (green) and cancer (red) were visualized as histograms in the outer bands of the Circos plot. Previously published nucleosome-bound cBAF cross-linking mass spectrometry data were combined and visualized as inner links on the Circos plot, where link thickness is proportional to the frequency of cross-links (the maximum frequency of cross-links is capped at 10 units). The Circos plot was made using the Circos software^135^.

Rolling averages of cancer and NDD mutational recurrence (missense and in-frame indels only) were calculated for BAF genes and visualized as a scatter plot with a regression line using the seaborn^136^ regplot function.

### NDD functional mutation analysis

Specific NDD residues predicted (by structural analysis) to disrupt buried residues (altering cavities), buried charged residues and hydrogen-bonds, BAF subunit or BAF module interaction, and BAF domain interaction were visualized in PyMol, with the disruptive NDD mutations indicated in red and putative interacting/proximal residues in blue or purple. Additionally, Missense 3D webserver with recombinant NCP-bound cBAF complex as input was used to assign functional consequences of some of these disruptive NDD mutations.

### NDD human versus yeast analysis

Select NDD residues in the integrated dataset were mapped to the recombinant NCP-bound cBAF complex (PDB: 6LTJ), yeast Swi/Snf (PDB:6UXW) and Snf2-nuclesome structures (PDB:5X0Y, 5X0X) were used to show that seemingly exposed residues on the cBAF structure are in fact buried by the brace helices in SMARCA2/A4 and that certain side-chain orientations in cBAF structure have different orientations in the yeast structures. SMARCA2/4 variant residues were mapped onto additional yeast Snf2-nucleosome structures (PDB:5Z3O, 5Z3U) to explore the open (ADP-bound) and closed (ADP-BeFx-bound) ATPase states and emphasize ATP and DNA interacting residues of the ATPase domain.

### Statistics and reproducibility

A hypergeometric test was used to determine the enrichment of genes of interest in a given gene set representing a specific biological process, molecular function, pathway or meaningful biological collection of genes. This analysis is more thoroughly described under NDD Gene Set Enrichment Analysis. OLS regression analysis was carried out using the default parameters in the seaborn regplot function.

No statistical method was used to predetermine sample size. Samples sizes for the hypergeometric test were determined using the standard procedure for GO, enrichment, or overrepresentation analysis.

Known duplicate samples or potentially duplicate samples from manual curation were excluded from analysis. Criteria for exclusion are thoroughly described under Curating mSWI/SNF gene NDD-associated variants. No other data were excluded from the analyses from variants collected from the aforementioned public or private databases. The experiments were not randomized. The investigators were not blinded to allocation during experiments and outcome assessment.

### Reporting summary

Further information on research design is available in the Nature Portfolio Reporting Summary linked to this article.

## Online content

Any methods, additional references, Nature Portfolio reporting summaries, source data, extended data, supplementary information, acknowledgements, peer review information; details of author contributions and competing interests; and statements of data and code availability are available at 10.1038/s41588-023-01451-6.

## Supplementary information

Reporting Summary

Supplementary Table 1 NDD-associated sequence variants from SPARK, SSC-ASC, DDD, DECIPHER, ClinVar, LOVD, literature review and 85 additional novel, previously unreported cases, including 72 novel variants.

Supplementary Table 2 Gene lists used for: 1) developmental disorder (DDG2P) genes, 2) curated chromatin regulators (this study), 3) SynGO gene list and gene symbol/id mappings (adapted from Koopmans et al.^83^ and 4) EpiFactor gene lists and complexes (adapted from Medvedeva et al.^82^.

Supplementary Table 3 mSWI/SNF gene information used for analysis: 1) mSWI/SNF gene list and Ensembl and UniProt IDs, 2) PFAM and InterPro domain annotations for mSWI/SNF genes, 3) predicted intrinsically disordered regions within mSWI/SNF genes, 4) ConSurf conservation details for NDD mutant residues, 4) summary of mappable mutations on recombinant and endogenous cBAF structures and 5) missense 3D predictions of nonpolar NDD-associated ACTB mutant residues.

Supplementary Table 4 Overlap of human cancer-and NDD- associated sequence variants with corresponding recurrence of missense mutations in NDD-only and shared NDD and cancer mutations.

## Data Availability

Public and private data can be accessed through their respective portals. Private data will require prior authorization. Data can be cleaned and normalized using any standard or well-established procedure for variant analysis or the procedures described in this paper, including referenced papers or procedures. The integrated, curated and de-duplicated data (to the best of our ability) are available in Supplementary Table 1. No additional data or intermediate results will be available upon request given the high manual burden to verify access to a variety of private portals, repositories and patients.

## Code availability

Variants were processed using well-established procedures described in the referenced papers. Datasets from diverse sources were integrated using a combination of code (to automate certain steps) and manual curation. Thus, the standalone code is not sufficient to regenerate the integrated dataset. Therefore, this code and intermediate results from dataset integration and curation is not available upon request. The code used for analysis and to generate figures is available under Creative Commons license through Zenodo at 10.5281/zenodo.8008632. Analyses were executed in Python (v3.7), R (v4.1.1), GraphPad Prism (v92.2), matplotlib(v3.3.1), circos (v0.69-9) and seaborn (v0.11.1).

PyMOL v2.4.0 was used to visualize structures. The Consurf online server was used for conservation analysis. Geneious Prime v2021.2.2 was used for multiple sequence alignmentss. The PolyPhen2 online server using the HumVar model was used to predict the severity/pathogenicity of the compiled NDD mutations. Unless otherwise noted, mutational counts, bar plots, pie charts, and Venn diagrams throughout were made using a combination of Python (v3.7), R (v4.1.1), GraphPad Prism (v92.2), matplotlib(v3.3.1) and seaborn (v0.11.1). The lollipop portion of the 2D schematics were created using the St. Jude PeCan Protein Paint software. Missense substitutions were visualized as a Sankey diagram using Google Charts. The Circos plot was made using the Circos software (v0.69-9). Missense substitutions were visualized as a Sankey diagram using Google Charts. The code used to process and visualize the data are available under the MIT license at Zenodo at 10.5281/zenodo.8008632.
