# Supplementary material for: Landscape of mSWI/SNF chromatin remodeling complex perturbations in neurodevelopmental disorders
Source: Nat Genet. 2023 Jul 27;55(8):1400–12. doi: 10.1038/s41588-023-01451-6 (PMC10412456; doi:10.1038/s41588-023-01451-6)
Supplement: Supplementary file 1 — Reporting Summary [file 41588_2023_1451_MOESM1_ESM.pdf]

nature portfolio

Corresponding author(s): Cigall Kadoch, Ph.D.

Last updated by author(s): 06/05/2023

Reporting Summary

Nature Portfolio wishes to improve the reproducibility of the work that we publish. This form provides structure for consistency and transparency in reporting. For further information on Nature Portfolio policies, see our Editorial Policies and the Editorial Policy Checklist.

Statistics

For all statistical analyses, confirm that the following items are present in the figure legend, table legend, main text, or Methods section.

n/a	Confirmed	
☐	☒	The exact sample size (*n*) for each experimental group/condition, given as a discrete number and unit of measurement
☒	☐	A statement on whether measurements were taken from distinct samples or whether the same sample was measured repeatedly
☐	☒	The statistical test(s) used AND whether they are one- or two-sided<br>*Only common tests should be described solely by name; describe more complex techniques in the Methods section.*
☒	☐	A description of all covariates tested
☒	☐	A description of any assumptions or corrections, such as tests of normality and adjustment for multiple comparisons
☒	☐	A full description of the statistical parameters including central tendency (e.g. means) or other basic estimates (e.g. regression coefficient) AND variation (e.g. standard deviation) or associated estimates of uncertainty (e.g. confidence intervals)
☐	☒	For null hypothesis testing, the test statistic (e.g. *F*, *t*, *r*) with confidence intervals, effect sizes, degrees of freedom and *P* value noted<br>*Give P values as exact values whenever suitable.*
☒	☐	For Bayesian analysis, information on the choice of priors and Markov chain Monte Carlo settings
☒	☐	For hierarchical and complex designs, identification of the appropriate level for tests and full reporting of outcomes
☒	☐	Estimates of effect sizes (e.g. Cohen's *d*, Pearson's *r*), indicating how they were calculated

Our web collection on statistics for biologists contains articles on many of the points above.

Software and code

Policy information about availability of computer code

Data collection	Data collection was done as described in the the Methods section of the manuscript and copied in the "Data" section below. Novel mSWI/SNF-NDD variants were identified as described in the "Novel Variant Collection" in the Methods of the manuscript, and summarized briefly in the "Human Research Participants" section in this document.
Data analysis	PyMOL v2.4.0 was used to visualize structures. The Consurf online server was used for conservation analysis. Geneious Prime v2021.2.2 was used for MSAs. The PolyPhen2 online server using the HumVar model was used to predict the severity/pathogenicity of the compiled NDD mutations. Unless otherwise noted, mutational counts, bar plots, heatmaps, and pie charts throughout were made using a combination of R (v4.1.1), GraphPad Prism (v9.2.0), and matplotlib (v3.3.1). The lollipop portion of the 2D schematics were created using the St. Jude PeCan Protein Paint software. Missense substitutions were visualized as a Sankey diagram using Google Charts. The circos plot was made using the circos software. The code used to process and visualize the data are available on request.

For manuscripts utilizing custom algorithms or software that are central to the research but not yet described in published literature, software must be made available to editors and reviewers. We strongly encourage code deposition in a community repository (e.g. GitHub). See the Nature Portfolio guidelines for submitting code & software for further information.

Data

Policy information about availability of data

All manuscripts must include a data availability statement. This statement should provide the following information, where applicable:

- Accession codes, unique identifiers, or web links for publicly available datasets
- A description of any restrictions on data availability
- For clinical datasets or third party data, please ensure that the statement adheres to our policy

Mutational Datasets: Open-access mutations publicly available on the DECIPHER database (https://www.deciphergenomics.org/; accessed June 22, 2022) (Firth et al., AJHG 2009) were used for broader chromatin gene analysis (Fig. 1c-d, S1k-l). The queried chromatin remodeling complex gene list (SWI/SNF, CHD, INO80, and ISWI) was manually curated from a literature review detailed below (Supplementary Table 2).

Chromatin Regulatory Gene Set (for Table S2): Chromatin remodeling complex (CRC) gene lists were curated from a variety of sources including HGNC gene groups SWI/SNF, INO80 (https://www.genenames.org/data/genegroup/#!/), as well as a literature review of the following: all CRCs– Sokpor et al., Front Neuro (2018), Hargreaves & Crabtree, Cell Res, (2011), mSWI/SNF– Mashtalir et al., Cell (2018), ISWI– Li et al, J Exp Clin Cancer Res (2021), CHD: Torrado et al., FEBS J 2017, and INO80: Sardiu et al, Sci Rep (2015), Giaimo et al, Epigenetics & Chromatin (2019), Frob & Wegner, Glia (2019), Willhoft & Wigley, Curr Opinion in Str Biol (2020), Feng et al, Cell Research (2018), Conaway & Conaway, Trends in Biochemical Sciences (2009). Histone modifier gene list was gathered from HISTome2 (Shah et al., Epigenetics & Chromatin (2020), Khare et al., Nucleic Acids Research (2012); http://www.actrec.gov.in/histome2/). Polycomb repressive complex genes were informed by Di Croce & Helin, Nat Struct Mol Biol (2013). DNA methylation regulatory genes were informed by Greenberg & Debroah Bourc'his, Nat Rev Mol Cell Biol (2019). Additional chromatin regulatory complexes were obtained from EpiFactor (Medvedeva et al., Database (2015); https://epifactors.autosome.org/protein_complexes). The full set of cBAF, PBAF, and ncBAF genes were included in the EpiFactor complexes if absent.

Curating mSWI/SNF gene NDD-associated variants: The set of rare inherited and de novo variants included data from three cohorts of individuals with autism spectrum disorders or other developmental disorders: the Simons SSC/ASC, SPARK, and DDD cohorts. Details about merging and de-duplicating the data are described in Fu et al. Briefly, duplicated samples were identified and excluded by IBD and other metadata, and the filtered samples were merged to provide a single unified set of de-duplicated de novo variants in autism spectrum disorders and other developmental disorders. The recurrence of NDD de novo variants across BAF genes and several gene sets of interest, including a curated set of chromatin remodelers, epigenetic modifiers, and synaptic genes were visualized with scatter plots and bar charts using matplotlib. The set of de novo variants and non-benign SNVs in DECIPHER were used for all summary calculations in figures 1 and S1, and for comparisons between the BAF genes, chromatin regulatory genes, epigentic modifier genes, and synaptic genes. The queried chromatin regulatory gene list was based on EpiFactor (https://epifactors.autosome.ru/genes; accessed September, 2 2021) (Medvedeva et al., Database 2015) updated to include all mSWI/SNF genes (Supplementary Table 2). The queried synaptic gene list was based on the SynGO gene list (https://www.syngoportal.org/ ; accessed September, 2 2021) (Koopmans et al., Neuron 2019). The development disorder DECIPHER gene list was based on the Development Disorder Genotype – Phenotype Database (DDG2P) genes on DECIPHER (accessed June 13, 2022).

A comprehensive list of single-nucleotide variant (SNV) and short in-frame shift mutations was compiled from an extensive literature review, the combined set of rare inherited and de novo variants from the Simons SSC/ASC, SPARK, DDD cohorts (the "combined cohort study"), the DECIPHER database of SNVs (https://www.deciphergenomics.org/), the merged set of de novo mutations from the DNM effort by McRae et al., NDD-associated ClinVar mutations (accessed 5/15/2021), NDD-associated variants from LOVD (LOVD v3.0 accessed 06/2022), and 85 previously unreported cases published in this study collected from the laboratories of Dr. Vergano and Dr. Santen.

First, the combined set of rare inherited and de novo variants were split into two: a set of rare inherited variants and a set of de novo variants. All rare inherited PTVs, in-frame indel variants, and de novo variants were included in the integrated dataset. Guided by the analysis in Fu et al., where missense variants with MPC scores (Missense badness, PolyPhen-2, and Constraint) of 1 or more were observed to confer moderate to strong levels of risk in developing autism, missense rare inherited variants with MPC scores >=1 were included in the integrated dataset. All other rare inherited variants from the combined cohort study were excluded. Then, samples were cross-referenced between the combined cohort study, DECIPHER database, and the DNM cohort of de novo mutations and identical variants from the same samples (using available sample IDs or aliases) were removed to de-deduplicate the data between these three cohorts / databases. Separately, a list of de novo variants in BAF genes across several other studies in the literature not covered previously by the cohorts used in DECIPHER and the combined cohort study (SSC/ASC, SPARK, DDD) were manually curated and de-duplicated to form the compiled set of mutations from the literature. Additionally, NDD-associated mutations from the LOVD database were compiled and filtered to include all PTV and in-frame indels and de novo/likely de novo missense variants. All benign/likely benign variants were excluded. The filtered set of LOVD variants and the manually curated variants from the literature were merged and de-duplicated based on sample IDs or aliases (if available) and study ID / reference (if sample IDs were not available). For shared variants between LOVD and the literature where it was not clear whether these variants were duplicates, only shared variants from the manually curated literature dataset were kept, effectively de-duplicating the data. Minimal overlap was assumed between the de-duplicated set of LOVD/literature variants and the de-duplicated set of SSC+ASC/SPARK/DDD/DECIPHER/DNM variants. These two sets were merged, followed by a round of manual curation to double check that as many duplicates or potential duplicates were removed during dataset integration. The set of 85 novel cases identified by Drs. Vergano and Santen were added to this merged dataset. In parallel, a curated set of ClinVar variants from samples with NDD-associated clinical features and unknown/likely pathogenic/pathogenic clinical significance was generated. Benign and likely benign ClinVar variants were excluded. Additionally, ClinVar variants submitted by GeneDx were excluded due to significant overlap with the comprehensive analysis of de novo mutations in NDD by Kaplanis et al. included in the DNM database of de novo mutations. Samples were de-duplicated between ClinVar and the LOVD/literature dataset using SCV codes wherever available. Finally, this de-duplicated ClinVar dataset was used to adjust the counts of the previously merged dataset of NDD-associated BAF mutations from the combined cohort study (SSC/ASC, SPARK, and DDD), DECIPHER SNVs, DNM, LOVD, and the literature. Note, it was difficult (and sometimes impossible) to track, match, and assign each filtered NDD-associated ClinVar SCV (submitted record for each variant) with the list of available sample IDs or aliases in the previously merged dataset. Thus, the total counts for each variant were adjusted to the total counts found in ClinVar (based on the number of submissions for each variant using SCV IDs) to eliminate the possibility of double counting if the ClinVar total count for a variant was more than the total count from the previously merged dataset. This procedure assumes submissions to ClinVar overlap entirely with the previously merged dataset, so it is possible the new merged dataset containing ClinVar variants might undercount some NDD-associated BAF variants. This integrated dataset was compared to gnomAD v3.1.2 to remove potential SNPs and other variants that occur frequently in a collection of healthy individuals. A more stringent MAF threshold of >=0.5% MAF was used to exclude potentially common variants in the integrated dataset. This final integrated dataset was manually checked once more to exclude potential duplicates and likely benign variants before freezing for all downstream analyses. A total of 2539 NDD-associated BAF variants are included in this dataset, including 85 novel cases, including 72 novel variants.

To standardize the data, all variants were remapped to the UniProt canonical BAF protein isoforms (see Supplementary Table S3) and duplicates, that could not be confirmed unique cases, were removed.

gnomAD variants of the general population were derived from the gnomAD v3 dataset (accessed 1/11/2021).

Cancer Datasets Cleaning and Compilation: PanCancer datasets from TCGA and cBioPortal were cleaned and compiled for all downstream analyses related to NDD vs Cancer comparisons.

The TCGA MC3 PanCancer dataset was used for NDD vs Cancer comparisons in FS1. Briefly, known SNPs were removed and BAF gene mutations were remapped to the canonical UniProt transcripts (see Supplementary Table S3). Missense, nonsense, and frameshift mutations were included, and all other mutations were excluded. This filtered set of mutations merged with the combined cohort study of NDD-associated mutations from the combined SSC/ASC, SPARK, and DDD cohorts. Total cancer missense, frameshift, and nonsense mutational recurrence was log normalized, compared to total de novo NDD-associated missense and PTV mutational recurrence for each gene, and visualized as a scatterplot using matplotlib with BAF genes indicated in red. The total proportion of NDD and Cancer missense and PTV mutations across the BAF genes were visualized as a grouped bar chart using matplotlib.

Mutations across BAF genes from the curated set of non-redundant studies in cBioPortal were compiled and filtered for NDD vs Cancer comparative analyses across the BAF genes. Briefly, the BAF mutations were remapped to the UniProt canonical BAF protein isoforms (see Supplementary Table S3). Missense, frameshift, nonsense, and in-frame indels were included, all other mutations were excluded. Additionally, duplicate mutations in patients with multiple samples were excluded. This filtered set of mutations from cBioPortal was used for downstream BAF Cancer vs NDD comparative analyses.

Domains annotations were compiled from PFAM, InterPro and the literature, and manually curated based on the AlphaFold EMBL-EBI structural predictions.

PDB models were obtained from RCSB and PDBDEV (PDB IDs: 6LTJ, 1RYU, 5B79, 7CYU; PDBDEV IDs: PDBDEV_00000056).

Data Availability
Public and private data can be accessed through their respective portals. Private data will require prior authorization. Data can be cleaned and normalized using any standard or well-established procedure for variant analysis or the procedures described in this paper, including referenced papers or procedures. The integrated, curated, and de-duplicated data (to the best of our ability) are available in Supplementary Table 1. No additional data or intermediate results will be available upon request given the high manual burden to verify access to a variety of private portals, repositories and patients. The code used to analysis and generate figures using variant data in Supplementary Table 1 is available under Creative Commons license through Zenodo at (doi: 10.5281/zenodo.8008632). Analyses were executed in Python (v3.7), R (v4.1.1), GraphPad Prism (v92.2), matplotlib(v3.3.1), circos (v0-69-9), and seaborn (v0.11.1).

Field-specific reporting

Please select the one below that is the best fit for your research. If you are not sure, read the appropriate sections before making your selection.

☒ Life sciences ☐ Behavioural & social sciences ☐ Ecological, evolutionary & environmental sciences

For a reference copy of the document with all sections, see nature.com/documents/nr-reporting-summary-flat.pdf

Life sciences study design

All studies must disclose on these points even when the disclosure is negative.

Sample size	2539 total variants in our dataset, integrated from the following sources: 1) ClinVar, 2) DECIPHER, 3) Literature review, 4) novel, previously-unreported variants, 5) LOVD, Simons Foundation Research Initiative (SFARI) datasets (including 6) SPARK, 7) SSC-ASC) and 8) the Deciphering Developmental Disorders (DDD) study. The collation of all of these data are found in Table S1, which includes source ID, cDNA, protein change, and other information).
Data exclusions	Potential variant duplicates, that could not be confirmed as unique cases, were removed. Non-mSWI/SNF variants within the Coffin-Siris Syndrome Registry dataset were excluded.
Replication	N/A
Randomization	N/A
Blinding	N/A

Reporting for specific materials, systems and methods

We require information from authors about some types of materials, experimental systems and methods used in many studies. Here, indicate whether each material, system or method listed is relevant to your study. If you are not sure if a list item applies to your research, read the appropriate section before selecting a response.

Materials & experimental systems

n/a	Involved in the study
☒	☐ Antibodies
☒	☐ Eukaryotic cell lines
☒	☐ Palaeontology and archaeology
☒	☐ Animals and other organisms
☐	☒ Human research participants
☒	☐ Clinical data
☒	☐ Dual use research of concern

Methods

n/a	Involved in the study
☒	☐ ChIP-seq
☒	☐ Flow cytometry
☒	☐ MRI-based neuroimaging

Human research participants

Policy information about studies involving human research participants

Population characteristics	This study analyzed data collected from human participants, however the covariate population characteristics are not included in the data reported as part of this study and were not used by the authors for analysis in this focused analysis. There are currently approximately 500 individuals in the Coffin-Siris syndrome Registry. Most are from the US but individuals from other countries have enrolled as well. Ages range from infancy to middle-age. The sex and age of individuals for whom novel variants are reported in this study are included in Supplementary Table 1.
Recruitment	Recruitment varied by institution.<br><br>The CSS registry: Eastern Virginia Medical School: Individuals are recruited to the registry through clinicians, social media, and patient foundations. Individuals complete an online consent form followed by a registry survey with phenotypic inquiries.<br><br>Leiden University Medical Center: No formal recruitment was performed for individuals. Individuals were identified through physician referrals which we have shared with their consent.
Ethics oversight	The Institutional Review Board of Leiden University Medical Center, Leiden, The Netherlands provided approval waivers for using de-identified data and publishing aggregated data (no: G18.098 and G21.129) without obtaining specific informed consent. The Coffin-Siris Syndrome Registry has been approved by the Eastern Virginia Medical School IRB (IRB# 15-03-EX-0058).

Note that full information on the approval of the study protocol must also be provided in the manuscript.
